# Immunotherapy against tau fragment diminishes AD pathology, improving synaptic function and cognition

**DOI:** 10.1186/s13024-025-00854-9

**Published:** 2025-05-27

**Authors:** Jie Xiang, Zhengjiang Qian, Ye Xi, Yanuo Wei, Guangxing Wang, Xia Liu, Zhi-Hao Wang, Zhentao Zhang, Shengxi Wu, Keqiang Ye

**Affiliations:** 1 Department of Neurobiology, Fourth Military Medical University, Xi’an, Shaanxi 710032 China; 2 Brain Cognition and Brain Disease Institute (BCBDI), Shenzhen Institutes of Advanced Technology (SIAT), Chinese Academy of Sciences (CAS), Shenzhen, Guangdong 518055 China; 3 Department of Laboratory Medicine, Sichuan Provincial People’s Hospital, University of Electronic Science and Technology of China, Chengdu, Sichuan 610072 China; 4 Department of Pathology and Laboratory Medicine, Emory University School of Medicine, Atlanta, GA 30322 USA; 5 Department of Neurology, Renmin Hospital of Wuhan University, Wuhan, 430060 China; 6 Department of Biology, Faculty of Life & Health Sciences, Shenzhen University of Advanced Technology (SUAT), Shenzhen, China

**Keywords:** TrkB receptors, Neurofibrillary tangles (NFTs), Tau N368 antibody, AEP, Alzheimer’s disease

## Abstract

**Background:**

Asparagine endopeptidase (AEP) is implicated in the pathogenesis of Alzheimer’s disease (AD) by cleaving Tau at residue N368, accelerating its hyperphosphorylation and aggregation. The Tau N368/t-Tau ratio in cerebrospinal fluid (CSF) serves as a superior biomarker compared to established biomarkers (p-Tau 181/217) for correlating with tau pathology and synaptic dysfunction in patients with AD, highlighting its diagnostic and therapeutic potential.

**Methods:**

We evaluated the therapeutic efficacy of a Tau N368-specific antibody in two mouse models: Tau P301S (tauopathy) and 3xTg (AD with Aβ/tau pathology). We conducted chronic intraperitoneal administration of the antibody to evaluate its effects on tau aggregation, synaptic integrity, and cognitive function. Neuropathological changes, synaptic plasticity (through electrophysiology), and behavioral outcomes were analyzed alongside Aβ pathology and neuroinflammation in 3xTg mice.

**Results:**

Treatment with the anti-Tau N368 antibody significantly diminished neurofibrillary tangles (NFTs) formed of hyperphosphorylated/truncated Tau in both models. Clearance of Tau restored BDNF/TrkB neurotrophic signaling, improved synaptic plasticity, and alleviated cognitive deficits. In 3xTg mice, this treatment also reduced Aβ deposition and neuroinflammation, resulting in enhanced learning and memory. Notably, the antibody’s effectiveness in alleviating both tau and Aβ pathologies indicates a potential interaction between these pathways.

**Conclusions:**

Targeting Tau N368 through immunotherapy alleviates tau-driven neurodegeneration, restores synaptic function, and improves accompanying Aβ pathology in AD models. Our results confirmed that Tau N368 is an exceptional biomarker and a promising therapeutic target, disrupting AD progression by addressing tau aggregation and its downstream effects.

**Supplementary Information:**

The online version contains supplementary material available at 10.1186/s13024-025-00854-9.

## Background

Alzheimer’s disease (AD) is the most prevalent dementia characterized by the presence of extracellular senile plaques, mainly consisting of aggregated amyloid-β (Aβ) and intraneuronal neurofibrillary tangles (NFTs), primarily composed of accumulated hyperphosphorylated and truncated tau, which are the cardinal pathological features in the brain. AD progresses in two stages. The first stage manifests the emergence and seeded propagation of abnormal Aβ and Aβ-associated pathologies, and the second stage involves complex, devastating changes that include NFTs, inflammation, vascular abnormalities, neurodegeneration, and, eventually, behavioral impairments [1]. In the two-stage model of AD [2, 3], Aβ aggregation initially drives the disease, but its relative influence diminishes concomitant with the emergence of a myriad of subsequent changes [4]. In the second stage, the disease appears to be independent of Aβ deposition [5].

The binding of brain-derived neurotrophic factor (BDNF) to its high-affinity receptor TrkB triggers Ras/Raf/MAPK, PI3K/Akt, and PLC-γ1 signal activation and is essential for the induction and maintenance of long-term potentiation (LTP) and long-term memory [6]. BDNF/TrkB neurotrophic signaling regulates neuronal development, differentiation, and survival, and deficient BDNF/TrkB activity underlies neurodegeneration in AD. For instance, BDNF and TrkB levels are reduced in the postmortem brain of AD patients [7–9]. Furthermore, BDNF levels are decreased at the mild cognitive impairment (MCI) stage of the disease and are correlated with cognitive functions [10]. BDNF and TrkB dysregulation contribute to AD neuropathology, most notably in hippocampal NPs (neuritic plaques) and NFTs [11]. In addition, increased serum pro-BDNF levels are associated with increased pTau staining in the hippocampus of AD patients [12]. Previously, we showed that the C-terminal tail of TrkB, to which PLC-γ1 binds, is involved in binding to Tau via its repeat domains (RDs; amino acids 256–368) [13]. Truncated Tau N368, cleaved by AEP (asparagine endopeptidase, also called δ-secretase), interacts with TrkB and blocks its neurotrophic signals, eliciting neuronal cell death. Remarkably, Tau N368 in CSF from human AD patients correlates with Tau PET images [14]. A longitudinal study revealed that Tau N368 in CSF may better capture Tau pathology and synaptic impairment than p-Tau 181 and total Tau, two well-established biomarkers [15]. Furthermore, the CSF Tau N368/total-Tau (t-Tau) ratio reflects cognitive performance and neocortical Tau better than p-Tau181 and p-Tau217 in cognitively impaired individuals [16]. These findings support that Tau N368 is critical for driving Tau pathology in AD pathogenesis. Notably, knockout of BDNF or TrkB induces AEP activation [17] and subsequent Tau N368 cleavage, triggering AD-like pathology and cognitive defects. Interestingly, blockade of Tau N368/TrkB complex with Tau repeat-domain 1 peptide diminishes the pathology [13]. Notably, deficiency in BDNF/TrkB neurotrophic activity enhances AEP expression by upregulating its upstream transcription factor C/EBPβ in AD [18, 19]. Therefore, altered BDNF and TrkB receptors are involved in AD pathology, and attenuating BDNF/TrkB signaling deficits may have therapeutic efficacy. Accordingly, TrkB agonists exhibit promising therapeutic effects in various AD mouse models [20–23].

The biphasic trajectory of AD pathogenesis has important implications for both treatment and prevention strategies. Aβ-immunotherapy significantly slows AD progression, indicating that a disease-modifying treatment for AD is feasible. However, the clinical benefit of these antibodies (lecanemab and donanemab) in trials is limited, and the disease still progresses in treated patients, albeit at a slower pace [24]. Hence, removal of aberrant Aβ in symptomatic AD patients is unlikely to be a cure for the disease, which begins in the brain 20–30 years before the onset of apparent cognitive impairment [25]. Because tau pathology is more strongly correlated with the degree of dementia than Aβ pathology, greater clinical efficacy may be achieved by clearing tau than by clearing Aβ aggregates in the later stages of the disease when cognitive impairments become evident [26]. Tau-targeted immunotherapy is a new direction after the failure of various inhibitors against Tau kinases or aggregates [27]. In the current study, we report that chronic Tau N368 antibody treatment substantially abrogates Tau pathology in both Tau P301S and 3xTg mouse models, alleviating cognitive deficits. Treatment strongly activates impaired BDNF/TrkB signaling, which is associated with elevated synaptic plasticity and decreased neuroinflammation. Therefore, this innovative immunotherapy provides a proof-of-concept of pathological Tau N368 clearance in AD treatment.

## Results

### mAbTau N368 specifically recognizes Tau N368 with high affinity

**Fig. 1:**
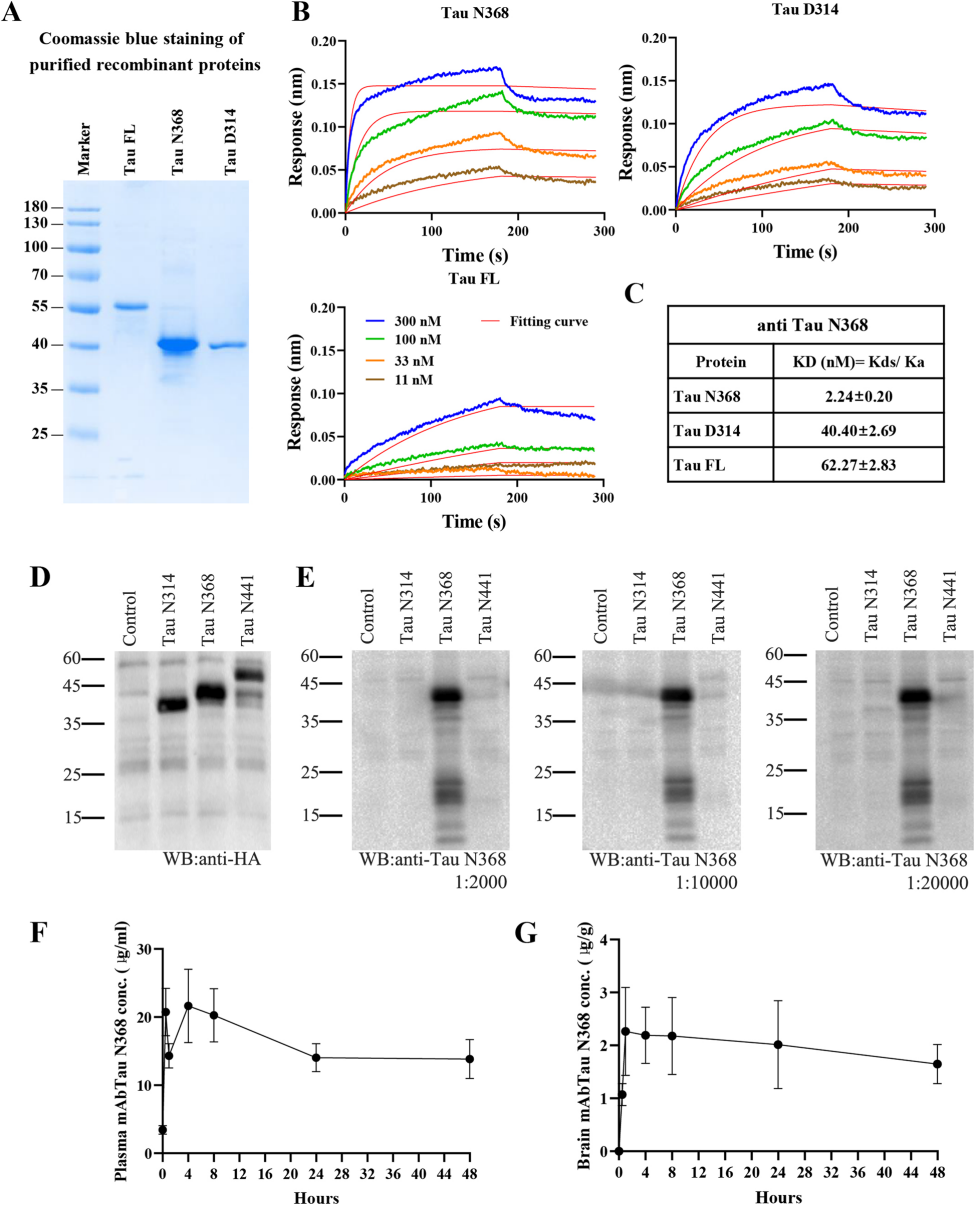
mAb Tau N368 specifically binds to Tau N368 and *iv vivo* PK. **A** Coomassie blue staining of purified recombinant proteins of Tau full length (Tau FL), Tau N368, and Tau D314. **B** Representative association/disassociation SPR curve of anti-Tau N368 interacting with purified proteins at different concentrations. **C** KD values of anti Tau N368 binding to Tau N368, Tau D314, and Tau FL. **D** IB verified HEK293 cells transfected HA-tagged Tau fragment expression. **E** IB analysis of the mAb Tau N368 detected Tau N368 bands in different dilutions; stocking concentration is 1 mg/ml. **F** Time course analysis of mAb Tau N368 levels in the plasma by ELISA, samples from 3-month C57 mice, *n* = 3 each time point, repetitive for 3 times. **G** Time course analysis of mAb Tau N368 levels in the brain by ELISA, samples from 3-month C57 mice, *n* = 3 each time point, repetitive for 3 times

Tau N368 is a neurotoxic pathological biomarker for AD [16]. It drives tau propagation in various tauopathy models [28–31]. To address whether Tau N368 immunotherapy demonstrates any therapeutic effects in the early stage of AD, we preliminarily evaluated whether Tau N368 antibody displays any effect of alleviating tau pathology. We selected about 4 months old P301S mice and treated them with polyclonal antibody via intraperitoneal (i.p.) injection with a dose of 10 mg/kg successively for 8-week, and we observed a significant decrease of Tau N368 levels in the mouse brains by Western blotting, as well as a decrease in phosphorylated tau expression, suggesting a favorable effect of the Tau N368 antibody (Fig S1A-B). Furthermore, we generated mouse mAbs against human 4R2N Tau360-368 peptide and identified an IgG1 clone. Surface plasmon resonance (SPR) assay showed that mAb Tau N368 specifically recognized the tau N368 antigen peptide with a binding constant (KD = Kd/Ka) value of 2.24 ± 0.20 nM (Fig. 1A-C). Immunoblot (IB) analysis showed that mAb bound to Tau N368 expressed in HEK 293 cells but not to full-length tau in wild-type or Tau D314 (Fig. 1D-E). We performed an in vivo PK study to quantitatively analyze the antibody tissue distribution. We administered tau N368 antibody via i.p. 10 mg/kg with WT mice and detected the antibody concentrations in the brain and plasma by ELISA at 30 min, 1 h, 4 h, 8 h, 1 day, 2 days (*N* = 3 each) after administration. The plasma concentration reached the highest at 4 h after injection, and the brain concentration slowly dropped 1 h after injection and remained relatively stable for 2 days (Fig. 1F-G).

### Anti-Tau N368 treatment eradicates Tau pathology in Tau P301S mice

**Fig. 2:**
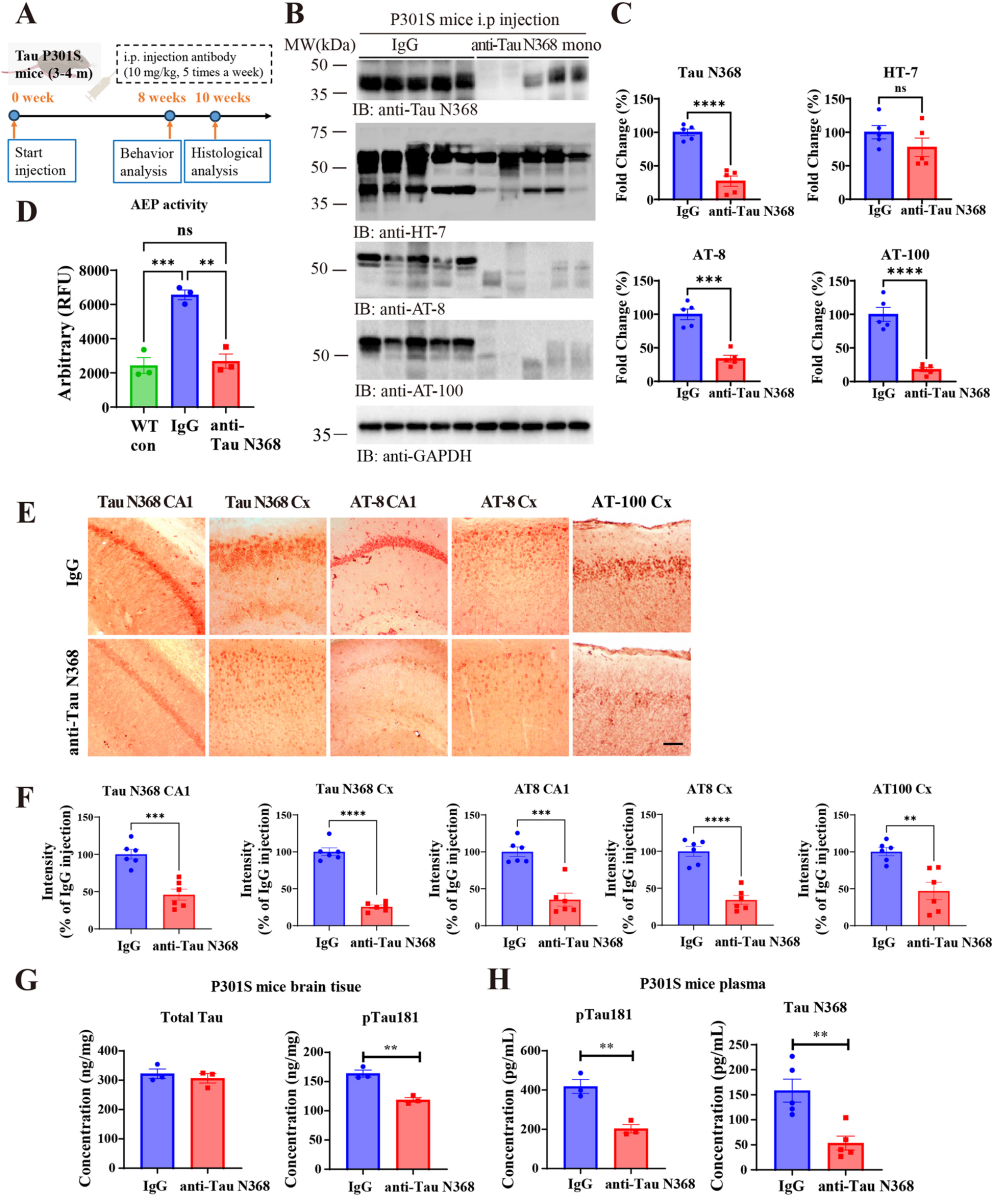
Anti-Tau N368 treatment inhibits Tau pathology in Tau P301S mice. See also Figure S1-S2. **A** Diagram showing the experimental schedule for anti-Tau N368 monoclonal treatment in Tau P301S mice. Mice aged 3–4 months were subjected to anti-Tau N368, which was used for behavioral tests and histological analysis. **B** Representative immunoblot images showing the effects of anti-Tau N368 treatment on the expression of soluble Tau pathology-related proteins in the brains of Tau P301S mice with or without anti-Tau N368 treatment. **C** Relative quantification of the protein levels in (**B**). The data are presented as the means ± s.e.m.; 5 mice in each group; *** *p* < 0.001, **** *p* < 0.0001 two-tailed Student’s t test compared with IgG. **D** AEP enzymatic activity in the brain tissue of Tau P301S mice with or without anti-Tau N368 treatment, age-matched WT control mice. The data are presented as the means ± s.e.m.; 3 mice in each group ** *p* < 0.01, *** *p* < 0.001, One-way ANOVA. **E** Immunohistochemistry (IHC) staining of Tau pathology-related proteins in the hippocampus and cortex of Tau P301S mice with or without anti-Tau N368 treatment. Scale bar, 50 μm. **F** Relative quantification of the signal intensity in (**E**). ** *p* < 0.01, *** *p* < 0.001, **** *p* < 0.0001compared with IgG. The data are presented as the means ± s.e.m; 4 sections from 6 mice in each group; two-tailed Student’s t test. **G** ELISA analysis of total Tau (left panel, including RIPA and sarkosyl extracted soluble tau) and pTau 181 (right panel) levels in the brain tissue of Tau P301S mice with or without anti-Tau N368 treatment. ** *p* < 0.01, compared with IgG, two-tailed Student’s t test.The data are presented as the means ± s.e.m.; 3 mice in each group. **H** Simoa analysis of pTau 181 (*n* = 3) and Tau N368 (*n* = 5) levels in the plasma of Tau P301S mice with or without anti-Tau N368 treatment. ** *p* < 0.01, compared with IgG, two-tailed Student’s t test

To explore whether Tau N368 monoclonal antibody could exert any immunotherapy efficacy, we injected ~ 4-month-old Tau P301S mice with 10 mg/kg antibody (i.p., once a day, five times a week) consecutively for 8 weeks (Fig. 2A), after the behavior assays that lasted 3 weeks, then sacrificed mice and performed various biochemical and staining tests at the age of 6 ~ 7-month-old. Immunoblotting (IB) revealed that Tau N368 and p-Tau (AT-8 and AT-100) were almost completely eradicated from the brain after chronic treatment (Fig. 2B and C). These effects were confirmed by immunofluorescence (IF) co-staining of hippocampal sections (Fig S2A&B). T22, a biomarker for aggregated Tau, was also highly reduced upon chronic anti-Tau N368 treatment (Fig S2C&D). Subsequently, the protease activity of AEP, an upstream protease that cleaves Tau into Tau N368 fragment, was significantly reduced by anti-Tau N368 (Fig. 2D). Consistent with the IB results, immunohistochemistry (IHC) confirmed that both Tau N368 and p-Tau were strongly decreased in the cortex and hippocampus after anti-Tau N368 treatment (Fig. 2E-H). To exclude the potential antigen masking effect from our Tau N368 antibody, we performed immunofluorescent staining by using different Tau N368 antibodies to verify Tau N368 expression in the hippocampus and obtained consistent results (Fig S2E&F). Concomitantly, p-Tau 181 in the brain was significantly decreased by this antibody, although total Tau levels remained unchanged (Fig. 2G). Remarkably, both p-Tau 181 and Tau N368 in the plasma were markedly decreased after treatment (Fig. 2H), indicating that Tau N368 is secreted not only in the CSF but also in the plasma. It may reside in both extracellular and intra-neuronal spaces. To further validate Tau pathology in P301S mice after antibody administration, we performed AV-1451 PET imaging to evaluate the mice’s Tau burden change in vivo. After Tau N368 antibody treatment, P301S mice showed a significant decrease in Tau tangles in vivo, consistent with the IHC staining results (Fig S2G-H).

### Anti-Tau N368 treatment restores synaptic plasticity and cognition in Tau P301S mice

**Fig. 3:**
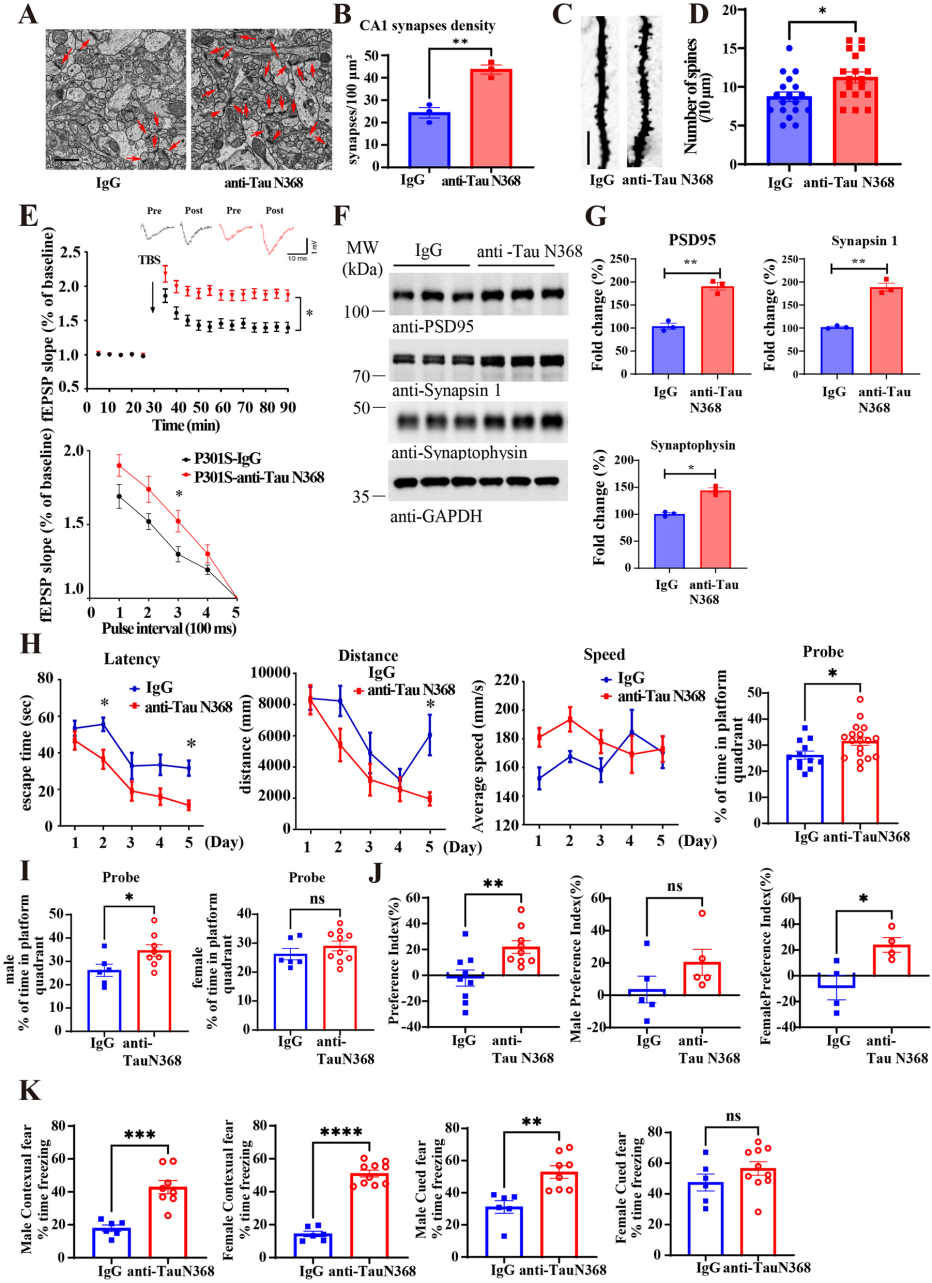
Anti-Tau N368 treatment improves synaptic function and cognitive deficits in Tau P301S mice. See also Figures S2 -S4. **A** Representative electron microscopy images of synaptic structures in the CA1 region. Red arrows indicate synapses (scale bar: 1 μm). **B** Quantification of synapse density in each group. (mean ± s.e.m.; *n* = 3 mice per group, 15 slices per mice. ** *p* < 0.01, two-tailed Student’s t test compared with IgG). **C** Representative image of Golgi staining was conducted on brain sections from CA1 regions of mice. Scale bar, 5 μm. **D** The quantified spines in **C**. (mean ± s.e.m.; *n* = 3 mice per group, 6 slices per mice; * *p* < 0.05, two-tailed Student’s t test compared with IgG). **E** Electrophysiology analysis. Treatment with the anti-Tau N368 antibody rescued LTP defects (upper panel) and increased the paired pulse (lower panel) ratio in P301S mice. (mean ± s.e.m.; *n* = 3 mice per group; * *p* < 0.05, two-tailed Student’s t test). The traces are representative fEPSPs recorded before and 60 min after theta-burst stimulation (TBS). **F** Representative immunoblot images showing the effects of anti-Tau N368 treatment on the expression of synaptic markers. **G** Relative quantification of protein levels. The data are presented as the means ± s.e.m; 3 mice in each group; *** *p* < 0.001, two-tailed Student’s t test compared with IgG. * *p* < 0.05, ** *p* < 0.01 compared with IgG. Two-tailed Student’s t test was used. **H–K**. Morris water maze (**H**), male and female probe (**I**), novel object recognition (**J**), and fear conditioning **(K**) tests showing the effect of anti-Tau N368 treatment on memory and cognitive behaviors in Tau P301S mice. **H** The Morris water maze parameters included latency, distance traveled, average speed, and number of probe trials. (mean ± s.e.m.; *n* = 12 mice in IgG group, *n* = 18 mice in anti-Tau N368 group; * *p* < 0.05, two-tailed Student’s t test and Two-way ANOVA). **I** The male and female mice showed different probe time in Morris water maze. (mean ± s.e.m.; group number was shown as plot dots; * *p* < 0.05, two-tailed Student’s t test). **J** The object recognition test showed that Tau N368 antibody-treated P301S mice spent less time exploring novel objects, the preference index is PI = (T_N_-T_F_)/(T_N_ + T_F_)) (data are means ± s.e.m.s, *n* = 9 per group, * *p* < 0.05, ** *p* < 0.01, two-tailed Student’s t test). **K** Fear conditioning test (mean ± s.e.m.; *n* = 12 mice in IgG group, *n* = 18 mice in anti-Tau N368 group; * *p* < 0.05, two-tailed Student’s t test)

Electron microscopy (EM) revealed that the number of synapses in the CA1 region of the hippocampus of Tau P301S mice was evidently increased upon Tau N368 immunotherapy (Fig. 3A and B). Golgi staining also demonstrated that the number of spine dendrites was significantly elevated (Fig. 3C and D). Consistent with the findings of dendrite and synapse augmentation, electrophysiology analysis of the brain sections showed that the fEPSP slope was strongly increased by Tau N368 antibody (Fig. 3E), indicating that long-term potentiation (LTP) and learning and memory were improved by Tau N368 immunotherapy. Consistent with the increase in synapses, the expression of synaptic biomarkers, including PSD95, synapsin 1, and synaptophysin, increased (Fig. 3F and G). The Morris water maze (MWM) test revealed that Tau N368 immunotherapy significantly reduced the latency and travel distance, although the swimming speeds were unaffected. Notably, Tau N368 antibody increased the amount of time spent in the platform quadrant, suggesting that Tau P301S mice exhibited improved spatial memory (Fig. 3H-I). A novel objective recognition assay revealed that Tau N368 antibody diminished the familiar object exploration time associated with increased object recognition, resulting in a prominent increase in the preference index (Fig. 3J). Fear conditioning tests revealed that chronic anti-Tau N368 treatment significantly alleviated cognitive dysfunction in Tau P301S mice (Fig. 3K). It is worth noting that the immunotherapy demonstrated better therapeutic efficacy in ameliorating cognitive dysfunctions in male mice than in female mice.

IF co-staining revealed that TUNEL signals in the hippocampus were significantly decreased by Tau N368 immunotherapy, indicating that pathological Tau clearance decreased neuronal cell death (Fig S3A&B). Interestingly, BDNF levels increased in the brain (Fig S3C). Correspondingly, p-TrkB Y816 was evidently activated, resulting in downstream p-Akt and p-Erk activation (Fig S3D&E). Thus, Tau N368 immunotherapy elevates BDNF concentrations in the brain and activates the BDNF/TrkB neurotrophic pathway, suppressing neuronal apoptosis. Moreover, quantification revealed that the levels of inflammatory cytokines, including TNFα, IL-1β and IL-6, were strongly decreased in the brains of Tau P301S mice after Tau N368 antibody treatment (Fig S4A). Consistently, microglial activation, indicated by IBA-1 immunostaining, was repressed, as was astrogliosis, as revealed by GFAP staining (Fig S4B-E). Hence, Tau N368 antibody treatment markedly ameliorates neuroinflammation in Tau P301S mice.

### Anti-Tau N368 treatment decreases Tau and Aβ pathologies in 3xTg mice

**Fig. 4:**
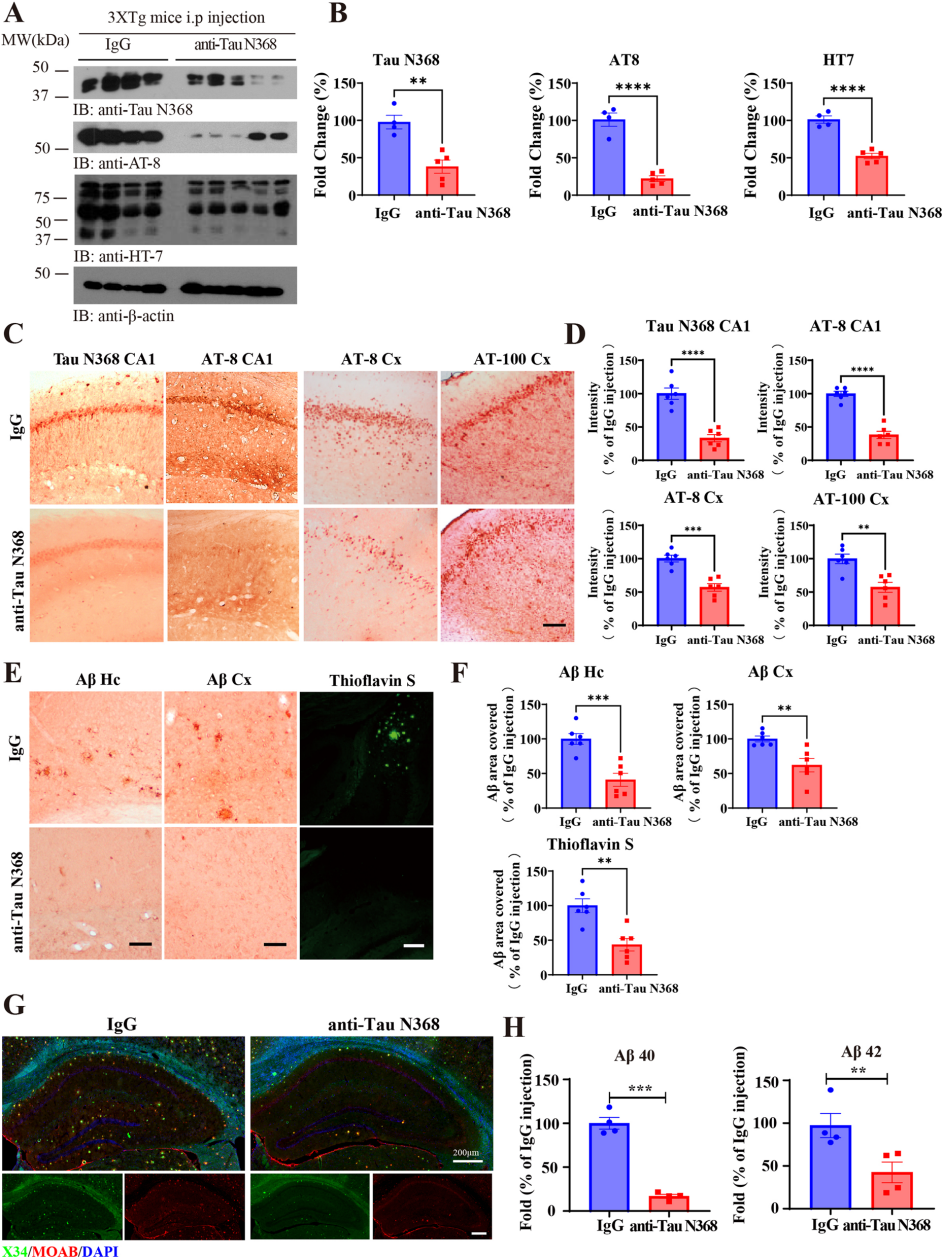
Anti-Tau N368 treatment inhibits Tau and Aβ pathology in 3xTg mice. **A** Representative immunoblot images showing the effects of anti-Tau N368 treatment on the expression of tau pathology-related proteins in the brains of 3xTg mice treated with anti-Tau N368. **B** Relative quantification of the protein levels in (**A**). * *p* < 0.05, ** *p* < 0.01, *** *p* < 0.001, compared with IgG. Two-tailed Student’s t test was used. The data are presented as the means ± s.e.m.; *n* = 4 (Control) or *n* = 5 (anti-Tau N368) mice in each group. **C** Immunohistochemistry (IHC) staining of Tau pathology-related proteins in the hippocampus and cortex of 3xTg mice with or without anti-Tau N368 treatment. Scale bar, 50 μm. **D** Relative quantification of the signal intensity in (**C**). ** *p* < 0.01, *** *p* < 0.001, **** *p* < 0.0001, compared with IgG.mean ± s.e.m.; *n* = 6 mice per group. **E** Immunohistochemistry (IHC) and thioflavin S (ThS) staining of Aβ plaques in the brains of 3xTg mice with or without anti-Tau N368 treatment. Scale bar, 50 μm. **F** Relative quantification of the Aβ area covered in (**E**). ** *p* < 0.01, *** *p* < 0.001, compared with IgG. The data are presented as the means ± s.e.m.; *n* = 6 mice in each group. **G** Whole hippocampus X34 and MOAB co-staining. Scale bar, 200 μm. **H** ELISA analysis of Aβ 40 (left panel) and Aβ 42 (right panel) levels in the brain tissues of 3xTg mice with or without anti-Tau N368 treatment. ** *p* < 0.01, compared with IgG. The data are presented as the means ± s.e.m.; *n* = 4 mice in each group

To further examine the therapeutic efficacy of Tau N368 antibody in treating tauopathies, we utilized a 3xTg AD mouse model. IB analysis revealed that the levels of Tau N368 and p-Tau (AT-8) and human Tau (HT-7) were obviously decreased after chronic Tau N368 antibody administration (Fig. 4A and B). IHC staining validated Tau N368, AT-8, and AT-100, with a prominent reduction in both the CA1 regions of the hippocampus and the cortex (Fig. 4C and D). Aβ acts upstream of Tau pathology by triggering its hyperphosphorylation and aggregation. Previously, we have shown that Tau N368 feeds back and stimulates the STAT1/BACE1 pathway, upregulating Aβ production [32]. Accordingly, Tau N368 antibody treatment robustly eliminated ThS-positive senile plaques in both the cortex and hippocampus (Fig. 4E-G). In agreement with these findings, Aβ40 and 42 in the brain were substantially reduced by anti-Tau N368 immunotherapy (Fig. 3H). Notably, Aβ burden reduction via an anti-Aβ monoclonal antibody is accompanied by decreased p-Tau species [33]. Similarly, anti-Tau N368 immunotherapy also diminished Aβ pathology in a 3xTg mouse model.

To further investigate whether Tau N368 was present in sarkosyl-soluble or insoluble tau aggregates, we extracted tau aggregates from P301S and 3xTg mice and conducted Western blotting. The blots revealed that Tau N368 existed in both soluble and insoluble forms. We noted a dramatic decrease in levels of sarkosyl-soluble and insoluble Tau N368, AT8, AT100, and total tau (Fig S5). These results demonstrated that the Tau N368 antibody can neutralize various tau aggregates. However, upon comparing the insoluble/soluble tau ratio, we observed a significant increase in the ratio of insoluble AT8 in P301S mice, indicating that AT8 tau primarily decreased in the soluble form. In contrast, the ratio in 3xTg mice showed no difference compared to the IgG group. These data suggested that the Tau N368 antibody has a different effect on P301S and 3xTg mice, potentially due to variations in their disease progression and tau aggregate characteristics.

Moreover, removing pathological Tau from 3xTg mice led to a reduction in AEP protease activity (Fig S6A). IB analysis also demonstrated p-TrkB/p-Akt/p-Erk signaling cascade activation in the brains of 3xTg mice upon Tau N368 immunotherapy (Fig S6B&C). Thus, these observations are consistent with our previous report that AEP cleaves the TrkB receptor, abolishing its neurotrophic signaling in AD pathogenesis [34]. In addition, Tau N368 antibody administration significantly diminished microglial activation and decreased neuroinflammation in 3xTg AD mice (Fig S7A&B). Additionally, ELISA revealed that inflammatory cytokines were decreased in the brains of 3xTg AD mice after Tau N368 antibody treatment (Fig S7C).

### Anti-Tau N368 treatment improves synaptic plasticity and cognition in 3xTg AD mice

**Fig. 5:**
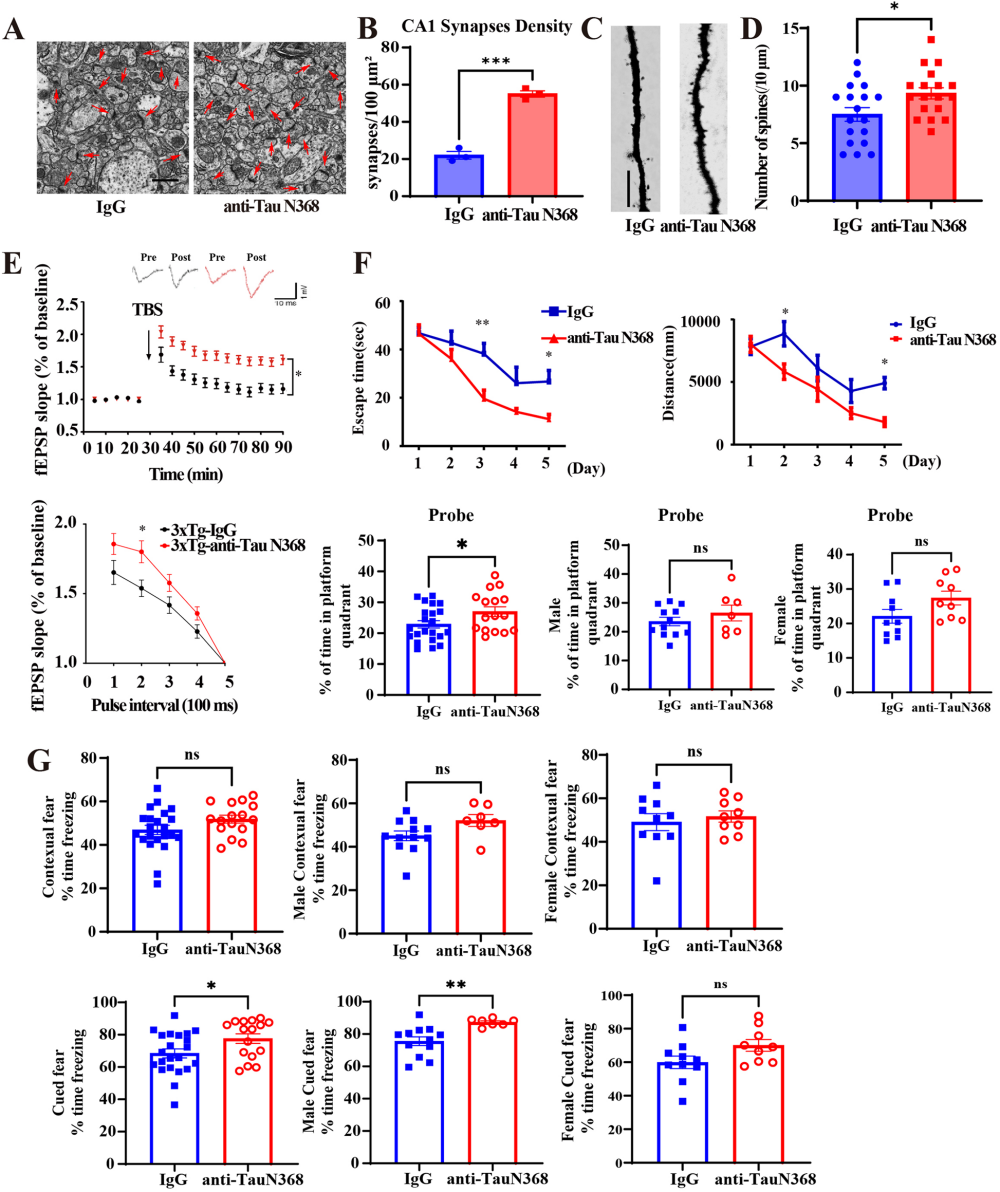
Anti-Tau N368 treatment improves synaptic and cognitive functions in 3xTg mice. See also Figures S5 and S6. **A** Representative electron microscopy images of synaptic structures in the CA1 region (left panel) in the hippocampal CA1 region of 3xTg mice with or without anti-Tau N368 treatment. Red arrows indicate synapses (scale bar: 2 μm). **B** Quantification of synapse density in (**A**). *** *p* < 0.001, compared with IgG. The data are presented as the means ± s.e.m.; *n* = 3 mice in each group, 15 sections per mice. **C** Golgi staining showing the dendritic spines from the apical dendritic layer of the CA1 region of 3xTg mice with or without anti-Tau N368 treatment. **D** Quantitative analysis of spine density in (**C**). * *p* < 0.05, compared with IgG. The data are presented as the means ± s.e.m.; *n* = 3 mice in each group, 6 slices per mice. **E** fEPSP slope (upper panel) and LTP of fEPSPs (lower panel) in each group. * *p* < 0.05, compared with IgG. The data are presented as the means ± s.e.m.; *n* = 3 mice in each group. **F** and **G** Morris water maze (**F**) and fear conditioning (**G**) tests showing the effect of anti-Tau N368 treatment on memory and cognitive behavior in 3xTg mice with or without anti-Tau N368 treatment. **F** The Morris water maze parameters included latency, distance traveled, and number of probe trials. (mean ± s.e.m.; *n* = 22 mice in IgG group; *n* = 16 mice in anti-Tau N368 group, * *p* < 0.05, ** *p* < 0.01, two-way ANOVA and unpaired t-test). **G** Fear conditioning test ((mean ± s.e.m.; *n* = 22 mice in IgG group; *n* = 16 mice in anti-Tau N368 group, * *p* < 0.05, ** *p* < 0.01, two-way ANOVA and unpaired t-test); *** *p* < 0.001, two-tailed Student’s t test)

EM studies and Golgi staining revealed that the number of synapses and spine dendrites was strongly increased in 3xTg AD mice by anti-Tau N368 (Fig. 5A-D). Electrophysiology revealed that LTP was significantly increased (Fig. 5E). Behavioral tests demonstrated that learning and memory were enhanced without altering swimming speeds in the MWM test (Fig. 5F). A fear conditioning assay also supported that Tau N368 antibody treatment improved the fear-freezing recovery duration (Fig. 5G). Therefore, Tau N368 immunotherapy significantly enhances synaptic plasticity and cognitive functions in 3xTg mice. To explore whether i.p. injected Tau N368 antibody penetrates the brain, we employed biotin-labeled control IgG and Tau N368 antibodies and found that biotinylated IgG and Tau N368 antibodies were brain permeable in both Tau P301S and WT mice, which were validated by IHC staining with streptavidin-HRP (Fig S8A). IF co-staining revealed that the antibodies readily entered MAP-2-positive neurons and GFAP-positive astrocytes in cultured organotypic brain slices (Fig S8B). Notably, the Biotin-Tau N368 antibody tightly colocalized with Tau N368 and AT100 in the brain (Fig S8C). Furthermore, Biotin-Tau N368 antibodies resided in LAMP1- and EEA1-positive lysosomes and endosomes (Fig S8D), suggesting that Tau N368 antibody may remove pathologically bound Tau N368 proteins or NFTs from these organelles for clearance.

### The anti-Tau N368 antibody stimulates uptake of Tau aggregates into microglia and reduces their seeding effect

**Fig. 6:**
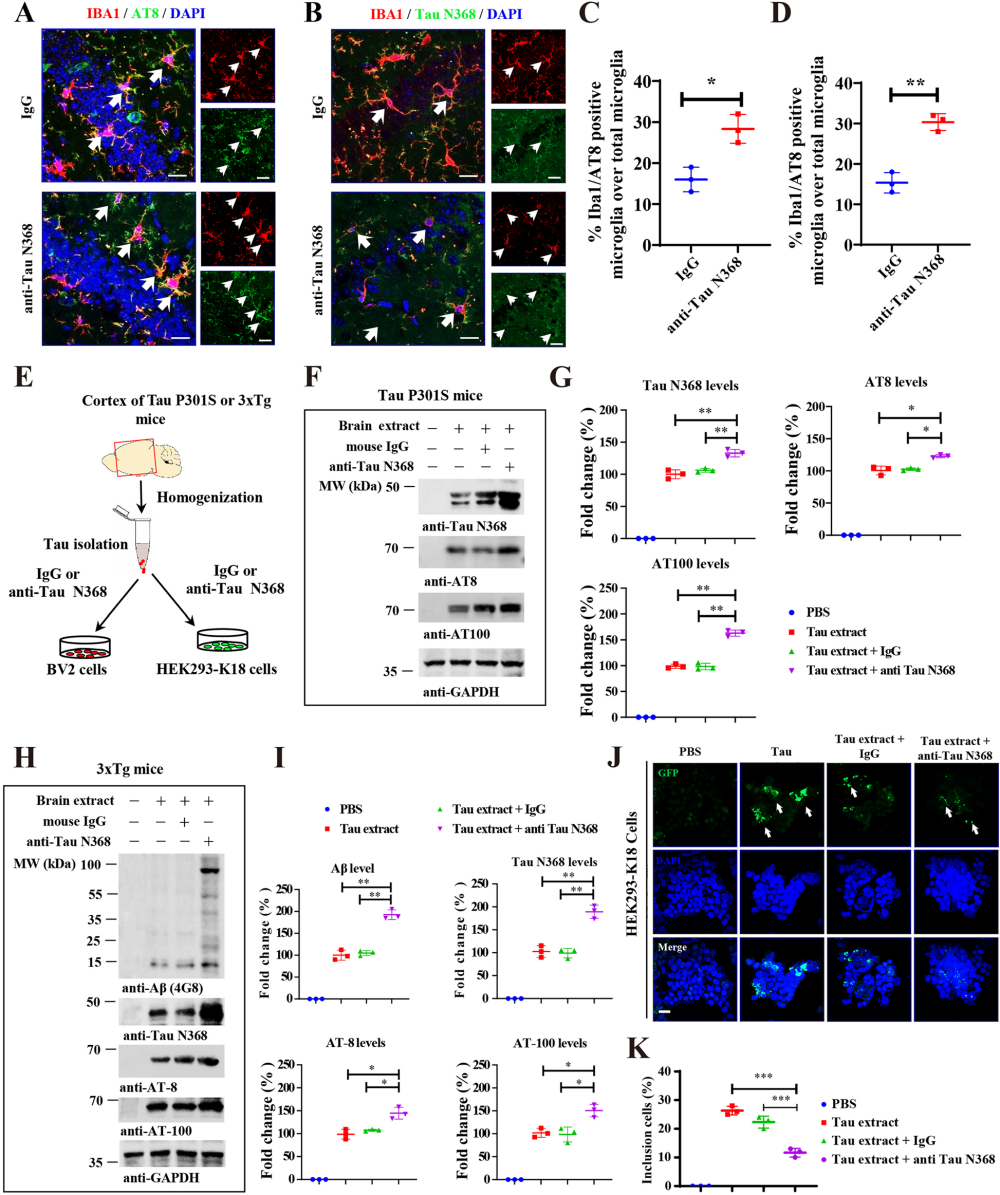
Anti-Tau N368 treatment elicits microglial phagocytosis and inhibits Tau propagation. See also Figure S7. **A** Representative immunofluorescence co-staining of AT8 and IBA1 in the hippocampus of Tau P301S mice with or without anti-Tau N368 treatment. Scale bar, 20 μm. **B** Representative immunofluorescence co-staining of Tau N368 and IBA1 in the hippocampus of Tau P301S mice with or without anti-Tau N368 treatment. Scale bar, 20 μm. **C** Quantification analysis of AT8 and IBA1 colocalization cells in (A). *p < 0.05 compared with IgG. The data are presented as the means ± s.e.m.; *n* = 3 mice in each group, 500 microglia counted in each mice. **D** Quantification analysis of Tau N368 and IBA1 colocalization cells in (B). **p < 0.01compared with IgG. The data are presented as the means ± s.e.m.; *n* = 3 mice in each group. **E** Diagram showing the experimental schedule for determining the effects of anti-Tau N368 on microglial and Tau aggregate activity. Tau aggregates were extracted from the cortex of Tau P301S or 3xTg mice and subjected to BV2 microglial and HEK 293-K18 cell isolation in the absence or presence of anti-Tau N368. **F** Representative immunoblot images showing the effects of anti-Tau N368 on BV2 microglial phagocytosis. **G** Relative quantification of Tau N368, AT8, and AT100 levels in the BV2 cells in (**F)**. *p < 0.05, ** *p* < 0.01, *** *p* < 0.001 compared with IgG; one-way ANOVA. The data are presented as the means ± s.e.m.; *n* = 3 mice in each group. **H** Representative immunoblot images showing anti-Tau N368 treatment increased BV2 phagocytosis of pathologic tau and Aβ in the brain extracts of 3xTg mice. **I** Relative quantification of Aβ, Tau N368, AT8, and AT100 levels in the BV2 cells in (F). *p < 0.05, ** *p* < 0.01 compared with IgG; The data are presented as the means ± s.e.m.; *n* = 3 mice in each group. **J** and **K** Tau was extracted from the brains of Tau P301S mice and subsequently incubated with control IgG or anti-Tau N368 antibody and then transduced into HEK293-K18 cells stably expressing GFP-tagged Tau RD (repeat domain). After 24 h of transduction, the insoluble Tau inclusions in the cells were imaged (**J**) and quantified (**K**) via microscopy. (*** *p* < 0.001 compared with IgG, one-way ANOVA). The data are presented as the means ± s.e.m.; *n* = 3 mice in each group.Sh

To assess whether anti-Tau N368 treatment promotes microglia phagocytosis in vivo, we co-stained with IBA1 and AT8 and showed that more AT8 signals co-localized in IBA1-positive microglia (Fig. 6A and C). As expected, most Tau N368 aggregates accumulated in the microglia (Fig. 6B and D), suggesting that Tau N368 antibody triggers phagocytosis of Tau aggregates into microglia. Western Blot showed increased TREM2 expression in P301S mice after Tau N368 treatment, indicating that microglia increase their response to Tau pathology [35] (Fig S9 A-B). To further explore the microglia’s response to immunotherapy, we also performed Tmem119 and CD68 staining to differentiate between activated and inactive microglia clearly. In comparison with the IgG group, anti-Tau N368 treatment mice exhibited higher expression of TMEM119 (marker of homeostatic microglia) but reduced CD68 (marker of disease-associated microglia) signals (Fig S9 C-F). These results indicate that Tau N368 treatment attenuates microglia reactivity and neuroinflammation in P301S mice. To investigate the potential molecular mechanism by which the Tau N368 antibody eliminates pathological Tau in tauopathy mouse models, we conducted an in vitro microglial uptake assay and seeding test. Initially, we isolated aggregated Tau inclusions from the cortex of Tau P301S mice or 3xTg mice and introduced them into the BV2 microglial cell line in the presence of control IgG or anti-Tau N368 antibody. IB analysis revealed that demonstrable Tau N368 was taken up by BV2 cells and associated with p-Tau in Tau fibrils isolated from Tau P301S mice, and the Tau N368 antibody prominently augmented this effect in comparison to that of the control IgG (Fig. 6E-G). Interestingly, when BV2 cells were exposed to 3xTg mice brain extracts, treatment of Tau N368 antibody increased microglial phagocytosis of both pathologic tau proteins and Aβ, indicating that microglia cells may play roles in Aβ reduction (F ig. 6H-I). These results indicate that microglia may engulf Tau aggregates in the brain and clear proteoglycans after the Tau N368 antibody binds to its targets. On the other hand, we introduced isolated Tau aggregates into HEK293-K18 cells, which were stably transfected with the Tau repeat domain (GFP-Tau RD). Conspicuously, these aggregates acted as seeds to elicit pronounced Tau aggregation in HEK293-K18 cells, which was significantly blocked by Tau N368 antibody compared to the effect of the control IgG (Fig. 6J-K), suggesting that anti-Tau N368 antibody strongly blocks Tau fibrils-induced Tau aggregation, which may indicate that Tau N368 antibody administration antagonizes the spread of Tau from one cell to the next cell in the brain.

## Discussion

AEP plays a crucial role in driving AD pathogenesis by cleaving both APP at residues N373 and N585 and Tau at residues N255 and N368, respectively, promoting Aβ accumulation as well as Tau hyperphosphorylation and aggregation [36, 37]. Notably, Tau N368 is elevated in AD and progressive supranuclear palsy (PSP) [36, 38]. This cleavage produces aggregation-prone tau fragments that propagate pathology. Clinical studies demonstrate that Tau N368 in CSF from human AD patients correlates with Tau PET images [14]. A longitudinal study shows that Tau N368 in CSF may better indicate Tau pathology and synaptic impairment than p-Tau 181 and total Tau, two established biomarkers [15]. Moreover, the CSF Tau N368/total-Tau (t-Tau) ratio reflects cognitive performance and neocortical Tau better than p-Tau181 and p-Tau217 in cognitively impaired individuals [16]. The previous studies also demonstrated that it drives Aβ upregulation by upregulating STAT1-BACE1 signaling [32]. These findings support the crucial role of Tau N368 in driving Tau pathology in AD.

The anti-tau N368 antibody specifically binds to the pathogenic form of Tau N368. Clinical evidence demonstrates that CSF levels of Tau N368 correlate more strongly with neurofibrillary tangle burden and synaptic dysfunction than with conventional biomarkers like p-Tau181, positioning it as a superior therapeutic target [14, 15]. In this study, chronic i.p. administration of Tau N368 antibody eradicated Tau N368 and Tau pathology in Tau P301S mice, leading to a reduction in p-Tau 181 in the brain and plasma. Following antibody treatment, the restoration of synaptic plasticity and cognitive function appears to be mediated through BDNF/TrkB signaling reactivation. Interestingly, after treatment with Tau N368, no difference is observed in total tau in PS19 mice. However, some reduction in total tau is noted in 3xTg mice. These two strains of mice demonstrate different levels of total Tau at various ages, along with distinct Tau pathologies. Conceivably, the efficacy of the Tau N368 antibody in eradicating total Tau varies across different Tauopathy models. Although the Tau N368 antibody binds explicitly to AEP-cleaved Tau truncate rather than FL Tau, the clearance of Tau N368 by its specific antibody alleviates neuroinflammation, which in turn reduces C/EBPβ activation, leading to a decrease in Tau expression. Moreover, Aβ accumulation occurs prior to Tau pathology in 3xTg mice. The Tau N368 antibody significantly removes Tau aggregates from 3xTg mice, which is associated with a reduction in Aβ as well. Since Aβ activates C/EBPβ [39], resulting in an increase in Tau expression, it is plausible that the decrease in Aβ due to the Tau N368 antibody in 3xTg mice also contributes to the reduction in Tau levels in these mice. Furthermore, we have shown that C/EBPβ acts as a repressor of BDNF [40]; thus, inactivating C/EBPβ likely leads to the upregulation of BDNF/TrkB signaling.

Notably, we observed that Aβ deposition was significantly decreased after Tau N368 immunotherapy in 3xTg mice, and AEP activity was inhibited after Tau N368 immunotherapy. Previously, we reported that AEP acts as a δ-secretase that cleaves APP at N373 and N585, promoting Aβ production. Hence, blockage of AEP activity results in less Aβ production [37]. In addition, Tau N368 activates STAT1, a BACE1 transcription factor that upregulates its expression, leading to Aβ escalation [32]. Hence, clearance of Tau N368 via immunotherapy diminishes Aβ in 3xTg mice. Moreover, this finding is consistent with a previous report that a Tau oligomer-specific monoclonal antibody decreases Aβ oligomer in Tg2576 mice, which is an APP (K670N/M671L) transgenic mouse model, which accumulates Aβ peptides, reversing memory deficits [41]. Therefore, these findings support the notion that Aβ and Tau mutually regulate each other, and the synergistic effect between them leads to subsequent loss of synapse and neurodegeneration [42–44]. Together, these findings strongly support that immunotherapy targeting Tau N368 may be a powerful pharmacological intervention strategy for treating AD.

An ideal immunoprevention strategy for AD should integrate findings from clinical trials with mechanistic insights from preclinical disease models to select promising antibodies, optimize the timing of intervention, identify early biomarkers, and mitigate potential side effects [45, 46]. Tau is likely a better target than Aβ once cognitive deficits manifest because the Tau burden correlates better with clinical impairments than Aβ [47]. Nevertheless, by the time the signs and symptoms of AD first appear clinically, damage to the brain is extensive and partially beyond repair; hence, complete restoration is unlikely, in line with the limited efficacy of Aβ antibody treatment trials [24, 48].

NFTs are intracellular and could limit the utility of anti-Tau antibodies as therapeutic agents. However, several groups have reported that antibodies, including anti-Tau antibodies, can enter neurons and that extracellular Tau might be important for the spread of Tau pathology [49, 50]. Mounting evidence shows anti-Tau antibodies cross the brain and enter neurons, primarily colocalizing with endosomal and lysosomal markers [51–54]. Using a Biotin-labeled Tau N368 antibody, we showed that this antibody penetrates the brain and resides in MAP2-positive neurons and GFAP-positive astrocytes. After entering brain cells, the antibodies may relocate bound pathological Tau to lysosomes and endosomes, eliciting Tau N368-containing NFTs for degradation and clearance. The entry of these antibodies into neurons is mainly receptor-mediated but can also occur via bulk endocytosis to some extent [55]. Recently, it has been shown that the cytosolic antibody receptor and E3 ligase TRIM21 could play a role in this effect on Tau pathology. Tau-antibody complexes are internalized into the neuronal cytoplasm, enabling TRIM21 engagement and protection against seeded aggregation. Antibody-mediated protection against Tau pathology is lost in mice lacking TRIM21. Thus, the cytosolic compartment provides a site of immunotherapeutic protection, which may aid in the design of antibody-based therapies for neurodegenerative disease [56].

A previous study showed that C/EBPβ, the key upstream transcription factor for AEP, in microglia plays a critical role in Tau pathology propagation in AD [57]. As expected, NFTs isolated from the brains of Tau P301S mice were engulfed by BV2 microglia, resulting in p-Tau (AT-8 and AT-100) escalation. These effects were robustly augmented by the Tau N368 antibody, suggesting that the Tau N368 antibody/NFT complex is highly internalized by BV2 microglia. Consequently, this antibody prominently blocks Tau aggregation in HEK-K18 cells via exogenously introduced NFT extracts, indicating that Tau N368 antibody might strongly repress Tau propagation. Hence, these findings shed light on the potential molecular mechanism underlying the therapeutic efficacy of Tau N368 antibody in two tauopathy mouse models.

Treatments are being developed to interfere with the aggregation process or promote Tau protein clearance. Several Tau-targeted passive immunotherapy approaches are being developed for treating tauopathies. At present, 12 anti-Tau antibodies have entered clinical trials, 7 of which are in clinical trials for primary tauopathies and AD. However, none of these seven agents have reached phase III [58, 59]. Various factors govern the efficacy of Tau antibodies, the most important of which are probably the epitope (normal or primarily pathological) and the site of action (extracellular and intracellular or only extracellular). It can be argued that extracellular clearance may be safer but less productive than intraneuronal clearance and/or sequestration to prevent the secretion and further spread of Tau pathology [26]. Our observations that Tau N368 is located in the brain and plasma indicate that it may be distributed extracellularly and intraneuronally. Tau N368 is a pathological trigger for Tau hyperphosphorylation and aggregation [36, 60]. Clinical studies directly comparing several different antibodies will be informative in identifying the most promising immunotherapeutic or immune-preventive Tau epitope. Therefore, identifying the properties of antibodies, such as affinity, immunoglobulin subtype, posttranslational modifications, and half-life in blood [61], should help to facilitate the design of next-generation humanized Tau N368 monoclonal antibodies and define the most suitable molecular target for immunotherapy in the clinic. A shift in the timing of intervention to the MCI stage or very early stages of AD has been a feature of many recent Aβ immunotherapy trials. Treatment before symptom onset might be feasible as diagnostic methods continue to improve. However, research to develop effective therapies once symptoms have progressed will always be needed [47]. Conceivably, for future investigations aiming to optimize the timing of immunoprevention, we need to determine whether humanized Tau N368 monoclonal antibodies could be administered intermittently and what the duration of treatment should be.

## Conclusion

The current study shows that Tau N368 antibody decreases Tau pathologies in mice with AD and restores BDNF/TrkB neurotrophic signaling and cognition. Using two different Tauopathy animal models, we chronically treated them with anti-Tau N368 via i.p. administration and found that this antibody effectively reduced AD pathology and increased synaptic plasticity, improving cognitive function. Mounting clinical studies support that Tau N368 truncate in CSF is a better biomarker than p-Tau 181 and p-Tau 217. Tau N368 acts as a seed to trigger Tau aggregation and propagation. Hence, this finding indicates that determining reduced Tau N368 levels in a patient’s serum or CSF may monitor target engagement in future clinical trials. Thus, this study provides proof-of-concept for Tau N368 immunotherapy against AD.

## Materials and methods

### Mice

P301S and 3xTg mice were obtained from the Jackson Laboratory (008169, 34830). All mice were housed in a room maintained at constant temperature and with a 12 h light/dark cycle. The protocol was reviewed and approved by the Institutional Animal Care and Use Committee of the Fourth Military Medical University and conformed to the Guide for the Care and Use of Laboratory Animals published by the National Institutes of Health (NIH). The mice age, sex, sample size of each experiment, treatments, et, were listed in the Supplementary Tables 1 and 2.

### Antibodies and reagents

The following antibodies and reagents were used in this study: anti-AT8 (Thermo Fisher, MN1020), anti-AT100, anti-T22 (Abcam, ABN454), anti-HT7 (Thermo Fisher, MN1000), anti-Tau N368 (Ye lab and ABN1703, Merck), anti-MOAB (abcam, ab126649), anti-Aβ (6E10, BioLegend, SIG-39300), anti-PSD95 (Cell Signaling Technology, #3409), anti-synapsin 1 (Cell Signaling Technology, #5297), anti-synaptophysin (Cell Signaling Technology, #36406), anti-Iba1 (VWR, 019 − 19741), anti-GFAP (Sigma‒Aldrich, G3893), anti-NeuN (Cell Signaling Technology, #94403), anti-pTrkB816 (Novus Biologicals, NBP1-03499), anti-pTrkB706 (Cell Signaling Technology, #4621), anti-TrkB (Cell Signaling Technology, #4606), anti-pAKT (Cell Signaling Technology, #4060), anti-AKT (Cell Signaling Technology, #9272), anti-pErk (Cell Signaling Technology, #4370), and anti-Erk (Cell Signaling Technology, #4621), anti-Trme2 (Cell Signaling Technology, #91068), anti-CD68 (abcam, ab125212), anti-TMEM119 (ab209064). Mouse TNFα, IL-1β and IL-6 ELISA kits (Beyotime), TUNEL apoptosis detection kit (Vazyme, A113-01), ThS (Sigma‒Aldrich, T1892), X34 (MCE, HY-D0236), Sarcosyl (Sigma, L7414), Aβ40 and Aβ42 ELISA kit (Thermo Fisher Scientific), and biotinylation kit (Thermo Fisher Scientific, 21935) were used.

### Cell culture and Tau antibody treatment

BV2 cells (a microglial cell line) were obtained from the American Type Culture Collection (ATCC, Manassas, VA). The cells were maintained in Dulbecco’s modified Eagle’s medium (DMEM; Gibco) supplemented with penicillin (20 unit/ml), streptomycin (20 mg/ml), and 5% heat-inactivated fetal bovine serum (FBS; HyClone). To observe the antibody-mediated phagocytic effect, 1 μg/ml antibodies (anti-Tau N368 or IgG) was added to the BV2 cells 4 h before treatment with 2 μg/ml tau extracts. After 24 h of treatment, the cells were harvested and examined for tau uptake by western blotting.

### Polyclonal antibody generation and purification

The Tau N368 antibody was derived from rabbits immunized with the recombinant peptide Ac-CITHVPGGGN-OH, and the eluted antibody was characterized by SDS‒PAGE, ELISA, and Western blotting. For antibody purification, a protein A column was prepared, the ascitic fluid was passed through the column, and the flow-through was collected. The bound antibody was eluted with 0.1 M citrate, pH 3.5; the pH was neutralized with 500 μl of Tris–HCl, pH 9.0.

### Monoclonal antibody generation and purification

BALB/c mice (2–3 months old) were immunized with 100 mg of Tau 368 peptide (Ac-CITHVPGGGN-OH) mixed with complete Freund’s adjuvant and boosted. At the end of immunization, spleen cells were harvested from mice to obtain spleen cell suspension and mixed with mouse myeloma cells SP2/0 to obtain fusion cells. Single pure clones were obtained by limiting dilution, and clones that showed positivity for Tau N368 protein in WB were selected. The selected clones were then negatively screened by WB involving other Tau recombinant proteins to obtain clones producing antibodies specifically against Tau N368. Finally, the selected hybridoma clonal cells were intraperitoneally injected into the BALB/c mice sensitized with paraffin oil. Ascites was collected 1 week post the injection, and antibodies were purified using a protein A column (HiTrap rProtein A FF column, GE Healthcare, Cat#17508001) according to the manufacturer’s instructions.

### Protein expression and purification

The human tau protein with full length, cleavage fragment at Tau N368 and Tau N314 were cloned into a pET-based vector with an N-terminal His6 tag fusion. The resulting plasmids were transformed into BL21 (DE3) *E. coli* strain (Transgene, Beijing). Protein expression was induced with 500 μM IPTG at OD600 0.5 overnight at 30 ℃. Cells were then pelleted, freeze-thawed, and resuspended in Lysis Buffer (20 μM Tris–HCl pH 8.0, 500 mM NaCl, 1 mM βME, and 20 mM imidazole) supplemented with 1 × protease inhibitor tablets, 1 mg/mL lysozyme, 2.5U/mL Turbo DNase (Life Technologies), and 2.5U/mL salt active nuclease (Sigma Aldrich). Lysed samples were then sonicated and clarified via centrifugation (18,000 × g for 1 h at 4 ℃), filtered with 0.45 μM PVDF filter. The samplee was applied to a Ni–NTA chromatography column (HiTrap, 5 mL GE Life Sciences), washed with 5 column volumes of Lysis Buffer, and 3 column volumes of Elution Buffer (20 μM Tris–HCl pH 8.0, 500 mM NaCl, 300 mM Imidazole). The samples were then subjected to gel filtration (Superdex 200 16/600, GE Life Sciences). Purified, eluted protein fractions were pooled, concentrated and frozen in PBS until use. All the purification procedure were performed using a AkTa system (GE Life Sciences).

### Surface plasmon resonance (SPR) assay

Binding assay of Tau N368 antibody with purified recombinant proteins (Tau FL, Tau N368 and Tau N314) was measured by biolayer interferometry on an Octet RED384 instrument (ForteBio, Inc.). The antibody of Tau N368 (5 μg/mL) were captured onto ProA biosensors and balanced with PBST (PBS with 0.02% Tween-20). The biosensors were then exposed to different concentration of purified recombinant proteins, followed by washing with PBST. Binding affinities (Kd) were calculated using the Blitz system software (ForteBio).

### mAb Tau N368 ELISA

Mice were administered with a single i.p. dose of mAb at 10 mg/kg. Blood samples were collected in tubes containing Na_2_EDTA anticoagulant from the mandibular vein 30 min, 1 h, 4 h, 8 h, 1 day, 2 days (*N* = 3 each) after the dose. Samples were then centrifuged at 4℃ at.

2,000 g for 15 min, and plasma was harvested and stored at 80℃ until analysis. Whole brain tissues were homogenized in lysis buffer containing 20 mM Tris–HCl (pH 7.4), 100 mM NaCl, 0.001% TritonX-100, phosphatase inhibitor cocktail and protease inhibitor cocktail (Roche). The homogenates were then centrifuged at 12,000 g for 15 min and the supernatant was subjected to ELISA analysis. The mAb was detected using an indirect enzyme-linked immunosorbent assay (ELISA). Ninety-six well ELISA plates were coated with 50 ng/well of Tau N368 peptide at 37℃ for 16 h. Plates were then washed with PBST (PBS containing 0.05% Tween 20) and blocked with 4% BSA at 37℃ for 2 h. After washing with PBST, plates were loaded with brain or plasma samples and incubated at 37℃ for 1 h. Plates were then washed 5 times with PBST and incubated with goat anti-mouse IgG1 HRP (1:2000, Abcam, 97240) at 37℃ for 1 h. Plates were washed 5 times with PBST, developed with ELISA TMB (Sigma) and read at 650 nm on a plate reader (THERMO Varioskan Flash). A standard curve was created by serial dilutions of mAb with known concentrations. The ELISA data was collected in a blind manner.

### Antibody administration

According to the characterization of the AD mice model pathology process, P301S and the 3xTg-AD mice received intraperitoneal (i.p.) injections of 10 mg/kg five times weekly for eight weeks, starting at approximately 4 months and 10 months of age, respectively (Supplementary Table 1 and 2) [62]. Mice were treated identically, but mice with IgG in saline were treated as controls. When performing behavior assays, the mice continued to be treated with antibody injections. The antibody administration was performed in a blinded manner.

### Aβ plaque staining

Amyloid plaques were labeled using thioflavin-S staining. The frozen brain sections were rinsed in distilled water and stained with 0.0125% thioflavin-S in 50% ethanol for 5 min. The sections were then washed with 50% ethanol and placed in distilled water before being covered with a glass cover using a mounting solution. Quantitative analysis of plaque areas was conducted utilizing ImageJ software.

### Isolation of insoluble Sarkosyl Tau aggregates in brain tissue

Sarkosyl-insoluble tau was extracted as described previously [63]. Briefly, 10-month-old Tau P301S mice or 3xTg mice were euthanized by cervical dislocation to preserve the metabolic environment of the brain and prevent artifacts that could alter the biochemical profiles of tau protein. Mouse brains were homogenized in ten volumes of Tris-buffered saline (TBS) buffer (50 mM Tris/HCl, pH 7.4, 274 mM NaCl, 5 mM KCl) containing protease and phosphatase inhibitors with a mechanical homogenizer. Centrifuge at 27,000 × g for 20 min at 4 ℃. The supernatant was retained as a soluble fraction of Tau. The pellet was homogenized with 5 vol. (v/w) of high salt/sucrose buffer (0.8 M NaCl, 10% sucrose, 10 mM Tris–HCl, pH 7.4, 1 mM EGTA, 1 mM PMSF). Centrifuge at 27,000 × g for 20 min at 4 ℃. The supernatant was adjusted to 1% Sarkosyl, incubated for 1 h at 37 ℃ on an orbital shaker, and centrifuged at 150,000 × g for 1 h at 4 ℃. The pellet was resuspended in TE buffer (10 mM Tris–HCl, pH 8.0, 1 mM EDTA) as a Sarkosyl insoluble fraction of Tau aggregates.

### Electron microscopy

Following deep anesthesia, the mice underwent transcardial perfusion with a solution containing 2% glutaraldehyde and 3% paraformaldehyde in phosphate-buffered saline (PBS). Subsequently, the hippocampal slices were further fixed in 1% OsO4 at low temperatures for 1 h. The samples were processed and analyzed using established protocols. Thin sections (90 nm) were stained with uranyl acetate and lead acetate and then observed at 100 kV using a JEOL 200CX electron microscope. Synaptic structures were identified based on the presence of synaptic vesicles and postsynaptic densities.

### Aβ ELISA

Mouse brain tissue was homogenized in lysis buffer and then centrifuged at 16,000 × g for 20 min at 4 °C. The resulting supernatant was subsequently examined using ELISA kits following the manufacturer’s guidelines.

### Small animal PET imaging

Mice PET experiments were performed as described previously [64, 65]. In brief, PET images were recorded on a high-resolution small animal scanning device (microPET) with a spatial resolution of 1.0 mm (SIAT). Brain emission scans were obtained in volumetric mode for the 20 min after an intravenous injection of 10–15 MBq of 18F-Flortaucipir (AV1451) in approximately 100 μL of saline into the vein of tail. The PET images were reconstructed by using ordered-subset expectation maximization (OSEM) with 16 subsets and 5 iterations. Standard uptake value (SUV) was obtained from a standardized target volume of interest, and was analyzed by Amide 1.0.4–1(San Diego, CA 92101) for scaling of 18F-AV1451 data.

### Simoa assay of blood samples

All the Simoa assays were performed on a Quanterix SR-X analyzer (Quanterix) as previously described [65]. Whole blood samples from the mice were centrifuged at 3000 × g for 10 min at 4 °C, and the plasma samples were collected and stored at − 80 °C until use. The concentrations of human pTau-181 were measured using the Simoa® pTau-181 Advantage V2 Kit (catalog #103714) according to the manufacturer’s instructions. Blood Tau N368 levels were measured according to the Quanterix homebrew protocol. The plasma samples were diluted from 1:6 to 1:20. All measurements were carried out in one round of experiments using the same batch of reagents.

### Immunofluorescence staining

Mice were subjected to deep anesthesia and subsequently perfused with ice-cold PBS, followed by 4% paraformaldehyde (PFA) for fixation. The entire brain was then carefully dissected, fixed in 4% PFA, dehydrated in 30% sucrose at 4 °C, embedded, and prepared for cryosectioning at a thickness of 20 µm. The cryosections were permeabilized and blocked in a solution containing 0.4% Triton X-100 and 5% normal bovine serum albumin (BSA) in PBS for 1 h at room temperature. Subsequently, the sections were incubated overnight at 4 °C with primary antibodies. Following PBS washes, the sections were exposed to secondary antibodies conjugated with Alexa Fluor 488 or 594 (Jackson ImmunoResearch Laboratories; 1:500) for 1 h at room temperature, stained with 4’,6-diamidino-2-phenylindole (DAPI) for 10 min, and then washed thrice with PBS. Finally, the coverslips were mounted onto glass slides using mounting medium, and the samples were visualized using a confocal microscope (FV1000, Olympus).

### Immunochemical staining

The mice were perfused with 4% paraformaldehyde, and the brains were postfixed and flash frozen in OCT. Twenty-micron-thick frozen sections were collected on slides. The sections were incubated with 3% H2O2 solution for 10 min to block endogenous peroxidase activity. After rinsing in PBS buffer, the sections were blocked with 5% BSA and 2% goat serum with 0.1% Triton X-100 PBS buffer for 1 h. Then, the sections were incubated with Tau N368 (mouse 1:500), AT-8 (1:500), AT-100 (1:500), Abeta (1:500), or Iba1 (1:200) overnight at 4 °C. After being washed with PBS thrice, the sections were incubated with biotinylated secondary antibodies for 1 h. Diluted streptavidin-HRP conjugates were applied for 30 min. Then, the DAB chromogen solution was used, and the tissue staining intensity was measured. The sections were stained with hematoxylin for nuclear visualization. Bright-field images were acquired with an Olympus BX-51 microscope. Male mice were used for all historical analysis. Two experienced pathologists assessed DAB-positive staining in a blind manner.

### Western blot analysis

Protein extraction from mouse brain tissue samples was conducted by utilizing lysis buffer supplemented with a mixture of protease inhibitors. The protein concentration in the samples was determined using a BCA protein assay kit (Thermo Fisher Scientific). Subsequently, equivalent amounts of protein were loaded onto SDS‒PAGE gels and transferred onto PVDF membranes. After blocking nonspecific binding with 5% nonfat milk for 1 h, the membrane was incubated with a specific primary antibody overnight at 4 °C, followed by incubation with a horseradish peroxidase–conjugated secondary antibody for 2 h at room temperature. The immunoblotting signals were detected using an enhanced chemiluminescence (ECL) kit (Yeasen, Cat. No., 36222ES60) in conjunction with a chemiluminescent imaging system (Bio-Rad). Quantification of the digital images was performed through densitometric analysis using ImageJ software.

### Transduction of Tau aggregates

HEK293-K18 cells that stably expressed the tau repeat domain were used to determine the seeding activity of the Tau extracts. Tau extracted from the cortex of the brain (2 μg/ml) was transfected into HEK293-K18 cells using Lipofectamine-3000 (Invitrogen) according to the manufacturer’s instructions. Eighteen hours later, the cells were fixed with 4% PFA in PBS for 10 min and then stained with DAPI for 5 min. Coverslips were mounted, sealed with nail polish, and placed at 4 °C before analysis under a Zeiss confocal imaging system (LSM 900). To quantify the percentage of cells positive for Tau inclusions, 10 fields were analyzed. The percentage of cells with inclusions was calculated based on the number of DAPI-positive nuclei.

### AEP activity assay

Tissue homogenates or cell lysates (10 μg) were incubated in 200 μl of reaction buffer (composed of 20 mM citric acid, 60 mM Na2HPO4, 1 mM EDTA, 0.1% CHAPS, and 1 mM DTT, pH 5.5) supplemented with 20 mM AEP substrate Z-Ala-Ala-Asn-AMC (Bachem). AMC was quantified through substrate cleavage by measuring the fluorescence at 460 nm using a plate reader at 37 °C in kinetic mode.

### Golgi staining

Mouse brain specimens were preserved in 10% formalin for 24 h and then in 3% potassium bichromate for 3 days under light-protected conditions, with daily solution changes. Then, the brains were placed in darkness in 2% silver nitrate for 24 h. Vibratome sections were obtained at 100 μm thickness, air-dried, dehydrated in ethanol, cleared in xylene, and coverslipped. Bright field images were acquired with an Olympus BX-51 microscope. The counting process was conducted by other researchers as blinded manner.

### Antibody biotinylation

The procedure followed the manufacturer’s protocol (Thermo Fisher Scientific, 21,935). Eight microliters of 9 mM Sulfo-NHS-LC-Biotin was added to 200 μl of 1 mg/ml Tau N368 antibody (in PBS buffer). The reaction mixture was incubated on ice for 3 h. A sufficient amount of biotinylated antibody was collected for further injection, and Zeba Spin Desalting Columns (40 K) were used to remove excess biotin.

### Organotypic brain slice culture

The organotypic hippocampal cultures used were described previously [66]. Briefly, postnatal day 5 P301S mice were decapitated, and the hippocampal region was dissected at 300 μm thickness by using a vibratome (Leica, VT1000). The slices were placed on cell culture plate inserts (Millipore) and incubated in DMEM supplemented with 15% FBS at 37 °C. Tau was extracted from the cortex of the brain (2 μg/ml) and added to the medium after culture for 2 days. After 24 h, 5 μg/ml biotinylated Tau N368 antibody or IgG was added to the medium. Then, the slices were fixed with 4% paraformaldehyde, and frozen sections were prepared for further experiments.

### Electrophysiology

This experiment was performed by an experienced physiologist in a blinded manner. P301S and 3xTg mice were anesthetized with isoflurane and decapitated, and their brains were placed in ice-cold artificial cerebrospinal fluid (aCSF) supplemented with 124 mM NaCl, 3 mM KCl, 1.25 mM NaH_2_PO_4_, 6.0 mM MgCl_2_, 26 mM NaHCO_3_, 2.0 mM CaCl_2_, and 10 mM glucose. Using a vibratome, the hippocampus was dissected and sliced into 400-μm thick transverse slices. Following incubation in a-CSF for 60–90 min, the slices were transferred to a recording chamber (RC-22 C, Warner Instruments) on the stage of an upright microscope (Olympus CX-31) and perfused at a rate of 3 ml/min with a-CSF (containing 1 mM MgCl_2_). A 0.1 MU tungsten monopolar electrode was used to stimulate the Schaffer collaterals. The field excitatory postsynaptic potentials (fEPSPs) in the CA1 stratum radiatum were recorded by a glass microelectrode filled with a-CSF with a resistance of 3–4 MUs. Field excitatory postsynaptic potentials (fEPSPs) were recorded in the CA1 stratum radiatum using a glass microelectrode filled with a-CSF. The stimulation output (Master-8; AMPI, Jerusalem) was controlled by the trigger function of an EPC9 amplifier (HEKA Elektronik, Lambrecht, Germany). fEPSPs were recorded under current-clamp mode. The data were filtered at 3 kHz and digitized at a sampling rate of 20 kHz using Pulse software (HEKA Elektronik). The stimulus intensity (0.1 ms duration, 10–30 mA) was set to evoke 40% of the maximum f-EPSP, and the test pulse was applied at 0.033 Hz. LTP of fEPSPs was induced by 3 theta-burst stimulation (TBS), which consisted of 4 pulses at 100 Hz and was repeated 3 times with a 200-ms interval). LTP magnitudes are expressed as the mean percentage of the initial slope of the baseline fEPSP.

### Morris water maze

P301S and 3xTg mice were trained in a round, water-filled tub (120 cm diameter) in an environment rich in extra maze cues as described previously [36]. Each subject was given 4 trials/day for 5 consecutive days with a 15-min intertrial interval. The maximum trial time was 60 s, and in cases where the subjects failed to reach the platform within the time limit, they were manually directed to it. After 5 days of task acquisition, a probe trial was presented. During this trial, the platform was removed, and the time spent in the quadrant where the platform was previously located was recorded. Latency and swim speed for all trials were assessed using Smart V3.0 software (RWD). Researchers conducted the whole procedure and analysis in a blinded manner.

### Contextual fear conditioning

Mice were placed in a fear conditioning apparatus composed of Plexiglass with a metal shock grid floor and allowed to explore the enclosure for 3 min. Subsequently, 3 conditioned stimulus (CS)-unconditioned stimulus (US) pairings were presented with a 1-min intertrial interval. The CS was composed of a 20 s, 85-dB tone, and the US consisted of 2 s of a 0.5-mA footshock co-terminated with each CS presentation. One minute following the last CS-US presentation, the mice were returned to their home cage. On the second day, a context test was conducted where the mice were placed back in the same chamber used for conditioning on the first day, and their freezing behavior was monitored using a camera and Coulbourn software without any shocks. On day 3, a tone test involving exposure to the CS in a new compartment was carried out. Initially, the animals were allowed to explore the new context for 2 min, after which the 85-db tone was played for 6 min, and the freezing behavior was recorded.

### Object recognition

Spatial memory was assessed within a white plastic chamber measuring 28 × 28 cm. Before the training session, all mice were acclimated to the behavioral room for a period of 30 min. On day 1, the mice were allowed to explore the chamber with patterns for 10 min. On day 2, the mice were introduced into the chamber with an object (a 7 cm tall glass flask filled with metal beads) placed adjacent to either patterned wall. The placement of the object was randomized. On day 3, the mice were reintroduced to the chamber with the object either in the same position as the previous exposure (familiar) or at a new location based on wall patterning. The time spent exploring the object, defined as having the nose within 1.5 cm of the object, was recorded for up to 15 min. Video analysis was conducted using SMART V3.0 (RWD). The preference index was calculated as PI = (T_N_-T_F_)/(T_N_ + T_F_). PI is the preference index, T_N_ is the time of a novel object, and T_F_ is the time of a familiar object.

#### Quantification and statistical analysis

All data are expressed as the mean ± s.e.m. from three or more independent experiments, and the significance level between the two groups was assessed with Student’s t-test. For more than two groups, one-way ANOVA followed by the LSD post hoc test was applied. A value of *p* < 0.05 was considered to indicate statistical significance.

## Supplementary Information

Supplementary Material 1.

Supplementary Material 2.

Supplementary Material 3.

Supplementary Material 4.

Supplementary Material 5.

## Data Availability

All data generated or analyzed during this study are included in this published article (and its supplementary information files).

## Abbreviations

AEP: Asparagine endopeptidase
AD: Alzheimer’s disease
CSF: Cerebrospinal fluid
NFT: Neurofibrillary tangles
Aβ: Amyloid-β
BDNF: Neurotrophic factor
MWM: Morris water maze
