# Supplementary material for: Immunotherapy against tau fragment diminishes AD pathology, improving synaptic function and cognition
Source: Mol Neurodegener. 2025 May 27;20:60. doi: 10.1186/s13024-025-00854-9 (PMC12117789; doi:10.1186/s13024-025-00854-9)
Supplement: Supplementary file 1 — Supplementary Material 1. [file 13024_2025_854_MOESM1_ESM.pdf]

1 st cohort

mouse lines P301S	NO	treatment	age of mice before immunot herapy	sex(M/F)	NOR	IHC for Tau	TUNEL	WB for Tau	TNF-a ELISA	IL-1beta ELISA	IL-6 ELISA	AEP assay	BDNF ELISA	Golgi staining
P301S	1	IgG	15w	M	Y	Y	Y							
P301S	2	IgG	15w	M	Y	Y	Y							
P301S	3	IgG	15w	M	Y	Y	Y							
P301S	4	IgG	15w	M	Y			Y	Y	Y	Y			
P301S	5	IgG	14w	M	Y			Y	Y	Y	Y			
P301S	6	IgG	15w	F	Y			Y	Y	Y	Y			
P301S	7	IgG	15w	F	Y			Y				Y	Y	
P301S	8	IgG	14w	F	Y			Y				Y	Y	
P301S	9	IgG	14w	F	Y									Y
	**total**			**9**	**9**	**3**	**3**	**5**	**3**	**3**	**3**	**2**	**2**	**1**
P301S	1	anti-Tau N368	15w	M	Y	Y	Y							
P301S	2	anti-Tau N368	15w	M	Y	Y	Y							
P301S	3	anti-Tau N368	15w	M	Y	Y	Y							
P301S	4	anti-Tau N368	14w	M	Y			Y	Y	Y	Y			
P301S	5	anti-Tau N368	14w	M	Y			Y	Y	Y	Y			
P301S	6	anti-Tau N368	14w	F	Y			Y	Y	Y	Y			
P301S	7	anti-Tau N368	14w	F	Y			Y				Y	Y	
P301S	8	anti-Tau N368	14w	F	Y			Y				Y	Y	
P301S	9	anti-Tau N368	14w	F	Y									Y
	**total**			**9**	**9**	**3**	**3**	**5**	**3**	**3**	**3**	**2**	**2**	**1**

2 nd cohort

mouse lines P301S	NO	treatment	age of mice before immunot herapy	sex(M/F)	MWM	FC	WB for TrkB	EM	TUNEL with NeuN	ePhy	AEP assay	BDNF ELISA	Golgi staining
P301S	1	IgG	15w	M	Y	Y	Y						
P301S	2	IgG	15w	M	Y	Y	Y						
P301S	3	IgG	15w	M	Y	Y	Y						
P301S	4	IgG	14w	M	Y	Y				Y			
P301S	5	IgG	14w	M	Y	Y				Y			
P301S	6	IgG	14w	M	Y	Y				Y			
P301S	7	IgG	15w	F	Y	Y	Y				Y	Y	
P301S	8	IgG	15w	F	Y	Y							Y
P301S	9	IgG	15w	F	Y	Y							Y
P301S	10	IgG	15w	F	Y	Y		Y					
P301S	11	IgG	14w	F	Y	Y		Y					

P301S	12	IgG	14w	F	Y	Y		Y								
	**total**			**12**	**12**	**12**	**4**	**3**	**0**	**3**	**1**	**1**	**2**			
P301S	1	anti-Tau N368	15w	M	Y	Y	Y									
P301S	2	anti-Tau N368	15w	M	Y	Y	Y									
P301S	3	anti-Tau N368	15w	M	Y	Y	Y									
P301S	4	anti-Tau N368	14w	M	Y	Y				Y						
P301S	5	anti-Tau N368	14w	M	Y	Y				Y						
P301S	6	anti-Tau N368	14w	M	Y	Y				Y						
P301S	7	anti-Tau N368	14w	M	Y	Y			Y							
P301S	8	anti-Tau N368	14w	M	Y	Y			Y							
P301S	9	anti-Tau N368	15w	F	Y	Y	Y				Y	Y				
P301S	10	anti-Tau N368	15w	F	Y	Y	Y									
P301S	11	anti-Tau N368	14w	F	Y	Y							Y			
P301S	12	anti-Tau N368	14w	F	Y	Y							Y			
P301S	13	anti-Tau N368	14w	F	Y	Y		Y								
P301S	14	anti-Tau N368	16w	F	Y	Y		Y								
P301S	15	anti-Tau N368	16w	F	Y	Y		Y								
P301S	16	anti-Tau N368	16w	F	Y	Y			Y							
P301S	17	anti-Tau N368	16w	F	Y	Y			Y							
P301S	18	anti-Tau N368	16w	F	Y	Y			Y							
	**total**			**18**	**18**	**18**	**5**	**3**	**5**	**3**	**1**	**1**	**2**			

3 rd cohort

**mouse lines P301S**	**NO**	**treatment**	**age of mice before immunotherapy**	**sex(M/F)**	**WB of synaptic markers**	**Tau ELISA**	**Tau 181 SIMOA**	**Tau 368 SIMOA**	**PET**	**TUNEL with NeuN**	**Microglia staining**	**Astrocyte Staining**	**Biotin-labeled in vivo**	**organotypic hippocampal slices**	**BV2 cell phagocytosis**	**microglia WB**
P301S	1	IgG	14w	M	Y	Y	Y	Y	Y							
P301S	2	IgG	14w	M	Y	Y	Y	Y	Y							
P301S	3	IgG	14w	M	Y	Y	Y	Y	Y							
P301S	4	IgG	14w	M				Y								
P301S	5	IgG	15w	M				Y								
P301S	6	IgG	15w	M						Y	Y	Y				
P301S	7	IgG	15w	M						Y	Y	Y				
P301S	8	IgG	15w	M						Y	Y	Y				
P301S	9	IgG	16w	M									Y			
P301S	10	IgG	16w	M									Y			
P301S	11	IgG	16w	M										Y		
P301S	12	IgG	16w	M										Y		
P301S	13	IgG	16w	M										Y		
P301S	14	IgG	16w	M											Y	Y
P301S	15	IgG	16w	M											Y	Y
P301S	16	IgG	16w	M											Y	Y
P301S	17	IgG	16w	F						Y			Y			
P301S	18	IgG	16w	F						Y			Y			
	**total**			**18**	**3**	**3**	**3**	**5**	**3**	**5**	**3**	**3**	**4**	**3**	**3**	**3**

P301S	1	anti-Tau N368	14w	M	Y	Y	Y	Y	Y							
P301S	2	anti-Tau N368	14w	M	Y	Y	Y	Y	Y							
P301S	3	anti-Tau N368	14w	M	Y	Y	Y	Y	Y							
P301S	4	anti-Tau N368	14w	M				Y								
P301S	5	anti-Tau N368	14w	M				Y								
P301S	6	anti-Tau N368	14w	M							Y	Y				
P301S	7	anti-Tau N368	14w	M							Y	Y				
P301S	8	anti-Tau N368	15w	M							Y	Y				
P301S	9	anti-Tau N368	15w	M									Y			
P301S	10	anti-Tau N368	15w	M									Y			
P301S	11	anti-Tau N368	15w	M										Y		
P301S	12	anti-Tau N368	15w	M										Y		
P301S	13	anti-Tau N368	15w	M										Y		
P301S	14	anti-Tau N368	15w	M												Y
P301S	15	anti-Tau N368	15w	M												Y
P301S	16	anti-Tau N368	15w	M												Y
P301S	17	anti-Tau N368	14w	F									Y			
P301S	18	anti-Tau N368	14w	F									Y			
	**total**			**18**	**3**	**3**	**3**	**5**	**3**	**0**	**3**	**3**	**4**	**3**	**0**	**3**

4 th cohort

**mouse lines P301S**	**NO**	**treatment**	**age of mice before immunotherapy**	**sex(M/F)**	**Sarkosyl extracted insoluble Tau WB**	**microglia staining**	**additional IF for Tau staining**
P301S	1	IgG	14w	M	Y		
P301S	2	IgG	14w	M	Y		
P301S	3	IgG	14w	M	Y		
P301S	4	IgG	14w	M		Y	Y
P301S	5	IgG	15w	M		Y	Y
P301S	6	IgG	15w	M		Y	Y
	**total**			**6**	**3**	**3**	**3**
P301S	1	anti-Tau N368	14w	M	Y		
P301S	2	anti-Tau N368	14w	M	Y		
P301S	3	anti-Tau N368	14w	M	Y		
P301S	4	anti-Tau N368	14w	M		Y	Y
P301S	5	anti-Tau N368	15w	M		Y	Y
P301S	6	anti-Tau N368	15w	M		Y	Y
	**total**			**6**	**3**	**3**	**3**
