# Supplementary material for: Immunotherapy against tau fragment diminishes AD pathology, improving synaptic function and cognition
Source: Mol Neurodegener. 2025 May 27;20:60. doi: 10.1186/s13024-025-00854-9 (PMC12117789; doi:10.1186/s13024-025-00854-9)
Supplement: Supplementary file 2 — Supplementary Material 2. [file 13024_2025_854_MOESM2_ESM.pdf]

1 st cohort

mouse lines 3xTg	NO	treatment	age of mice before immunotherap	sex(M/F)	MWM	FC	WB for Tau	WB for TrkB	IHC for Tau	Abeta IHC	Iba1 IHC	AEP assay	TNF-a ELISA	IL-1beta ELISA	IL-6 ELISA
3xTg	1	IgG	10m	M	Y	Y	Y	Y							
3xTg	2	IgG	10m	M	Y	Y	Y	Y							
3xTg	3	IgG	10m	M	Y	Y			Y	Y	Y				
3xTg	4	IgG	9m3w	M	Y	Y			Y	Y	Y				
3xTg	5	IgG	9m3w	M	Y	Y			Y	Y	Y				
3xTg	6	IgG	10m	F	Y	Y	Y	Y							
3xTg	7	IgG	10m	F	Y	Y	Y	Y							
3xTg	8	IgG	10m	F	Y	Y						Y	Y	Y	Y
3xTg	9	IgG	9w3w	F	Y	Y						Y	Y	Y	Y
3xTg	10	IgG	9m3w	F	Y	Y						Y	Y	Y	Y
	**total**			**10**	**10**	**10**	**4**	**4**	**3**	**3**	**3**	**3**	**3**	**3**	**3**
3xTg	1	anti-Tau N368	10m	M	Y	Y	Y	Y							
3xTg	2	anti-Tau N368	10m	M	Y	Y	Y	Y							
3xTg	3	anti-Tau N368	10m	M	Y	Y	Y	Y							
3xTg	4	anti-Tau N368	9w3w	M	Y	Y			Y	Y	Y				
3xTg	5	anti-Tau N368	9m3w	M	Y	Y			Y	Y	Y				
3xTg	6	anti-Tau N368	9m3w	M	Y	Y			Y	Y	Y				
3xTg	7	anti-Tau N368	10m	F	Y	Y	Y	Y							
3xTg	8	anti-Tau N368	10m	F	Y	Y	Y	Y							
3xTg	9	anti-Tau N368	10m	F	Y	Y						Y	Y	Y	Y
3xTg	10	anti-Tau N368	9m3w	F	Y	Y						Y	Y	Y	Y
3xTg	11	anti-Tau N368	9m3w	F	Y	Y						Y	Y	Y	Y
	**total**			**11**	**11**	**11**	**5**	**5**	**3**	**3**	**3**	**3**	**3**	**3**	**3**

2 nd cohort

mouse lines 3xTg	NO	treatment	age of mice before immunotherap	sex(M/F)	MWM	FC	EM	Golgi staining	ePhy
3xTg	1	IgG	9m3w	M	Y	Y	Y		
3xTg	2	IgG	9m3w	M	Y	Y	Y		
3xTg	3	IgG	9m3w	M	Y	Y			Y
3xTg	4	IgG	10m2w	M	Y	Y			Y(FAILED)
3xTg	5	IgG	10m2w	M	Y	Y			Y(FAILED)
3xTg	6	IgG	10m2w	M	Y	Y			Y
3xTg	7	IgG	10m2w	M	Y	Y			Y

3xTg	8	IgG	9m3w	F	Y	Y	Y(FAILED)		
3xTg	9	IgG	9m3w	F	Y	Y		Y(FAILED)	
3xTg	10	IgG	10m2w	F	Y	Y		Y	
3xTg	11	IgG	10m2w	F	Y	Y		Y	
3xTg	12	IgG	10m2w	F	Y	Y		Y	
	**total**			**12**	**12**	**12**	**2**	**3**	**3**
3xTg	1	anti-Tau N368	10m	M	Y	Y	Y		
3xTg	2	anti-Tau N368	10m	F	Y	Y	Y		
3xTg	3	anti-Tau N368	10m	F	Y	Y		Y	
3xTg	4	anti-Tau N368	10m	F	Y	Y		Y	
3xTg	5	anti-Tau N368	10m	F	Y	Y		Y	
	total			**5**	**5**	**5**	**2**	**3**	**0**

3 rd cohort

**mouse lines 3xTg**	**NO**	**treatment**	**age of mice before immunotherap**	**sex(M/F)**	**EM**	**ePhy**
3xTg	1	IgG		M	Y	
	**total**			**1**	**1**	**0**
3xTg	1	anti-Tau N368	9m3w	M	Y	
3xTg	2	anti-Tau N368	9m3w	M		Y
3xTg	3	anti-Tau N368	9m3w	M		Y
3xTg	4	anti-Tau N368	9m3w	M		Y
	total			4	1	3

4 th cohort

**mouse lines 3xTg**	**NO**	**treatment**	**age of mice before immunotherap**	**sex(M/F)**	**MOAB/X 34**	**BV2 cell phagocytosis**
3xTg	1	IgG	10m	M	Y	
3xTg	2	IgG	10m	M	Y	
3xTg	3	IgG	10m	M	Y	
3xTg	4	IgG	10m2w	M		Y
3xTg	5	IgG	10m2w	M		Y
3xTg	6	IgG	10m2w	M		Y
	total			6	3	3
3xTg	1	anti-Tau N368	10m	M	Y	
3xTg	2	anti-Tau N368	10m	M	Y	
3xTg	3	anti-Tau N368	10m	M	Y	
3xTg	4	anti-Tau N368	10m	M		Y
3xTg	5	anti-Tau N368	10m2w	M		Y
3xTg	6	anti-Tau N368	10m2w	M		Y
	total			6	3	3

5 th cohort mouse lines 3xTg	NO	treatment	age of mice before immunotherap	sex(M/F)	IHC for Tau	Abeta IHC
3xTg	1	IgG	9m3w	M	Y	Y
3xTg	2	IgG	9m3w	M	Y	Y
3xTg	3	IgG	9m3w	M	Y	Y
	total			3	3	3
3xTg	1	anti-Tau N368	9m3w	M	Y	Y
3xTg	2	anti-Tau N368	9m3w	M	Y	Y
3xTg	3	anti-Tau N368	9m3w	M	Y	Y
	total			3	3	3
