# Supplementary material for: Immunotherapy against tau fragment diminishes AD pathology, improving synaptic function and cognition
Source: Mol Neurodegener. 2025 May 27;20:60. doi: 10.1186/s13024-025-00854-9 (PMC12117789; doi:10.1186/s13024-025-00854-9)

Extra Added 3xTg mice brain samples for Beta-Amyloid immunostaining

CA1 region 4G8

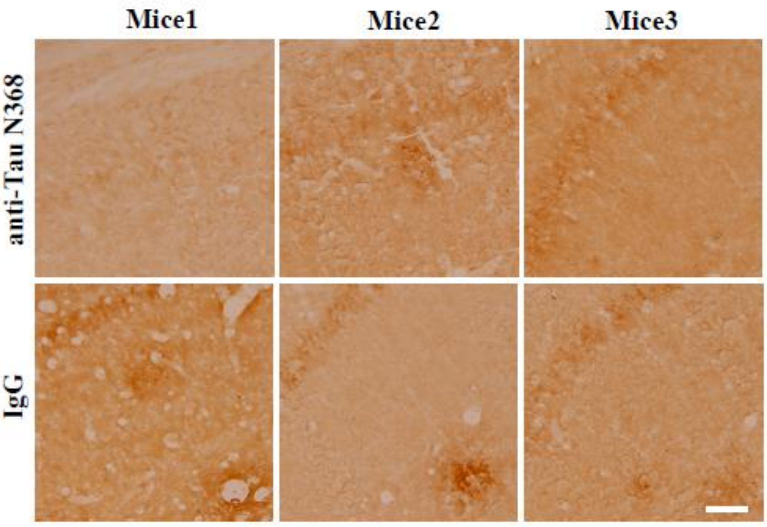

Cortex region 4G8

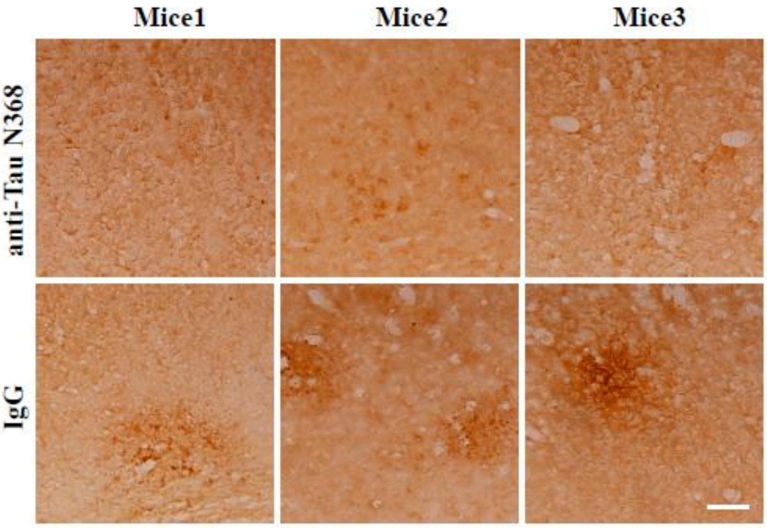

Extra Added 3xTg mice brain samples for Tau immunostaining

CA1 region AT8

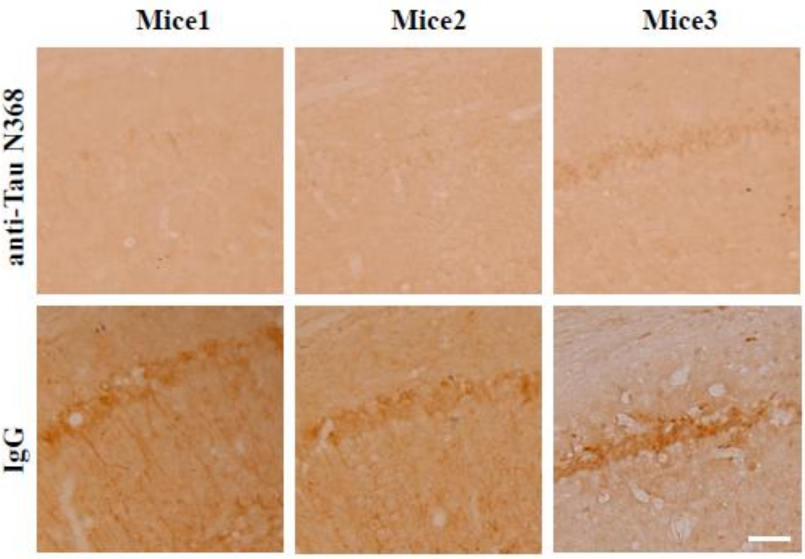

Cortex region AT8

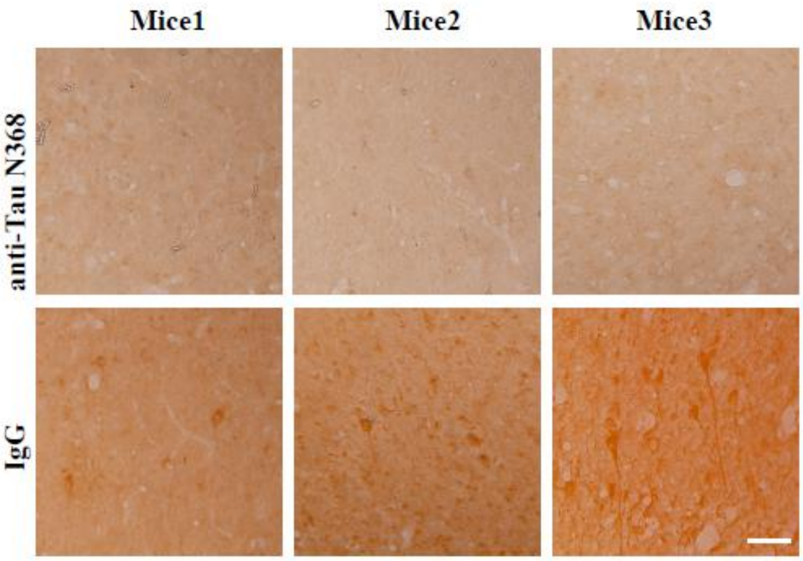

Extra Added 3xTg mice brain samples for Tau immunostaining

CA1 region AT100

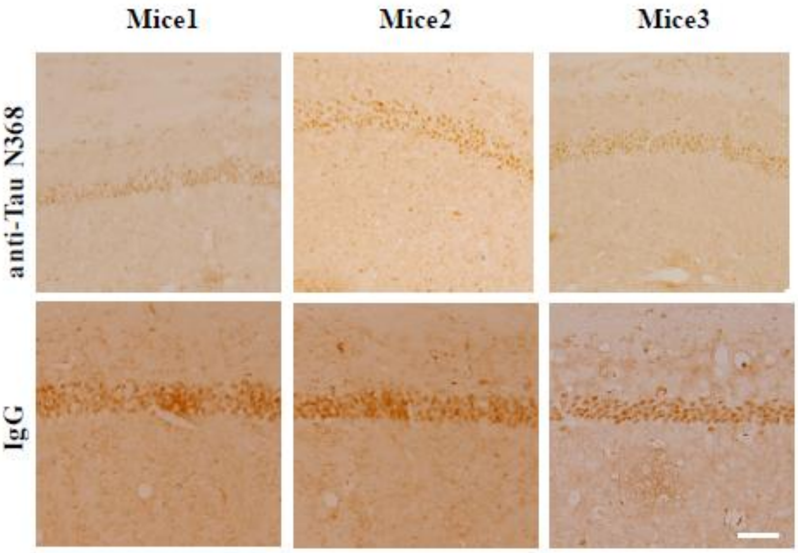

Cortex region AT100

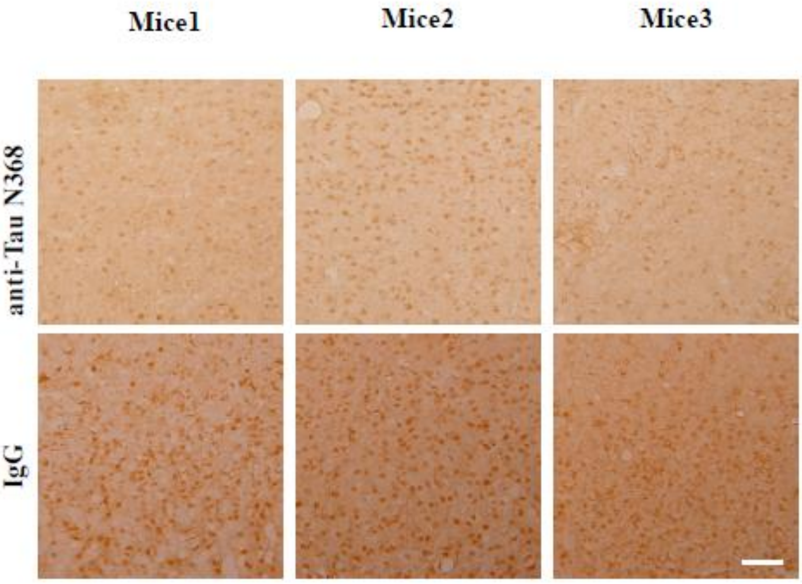

Extra Added 3xTg mice brain samples for Tau immunostaining

CA1 region Tau N368

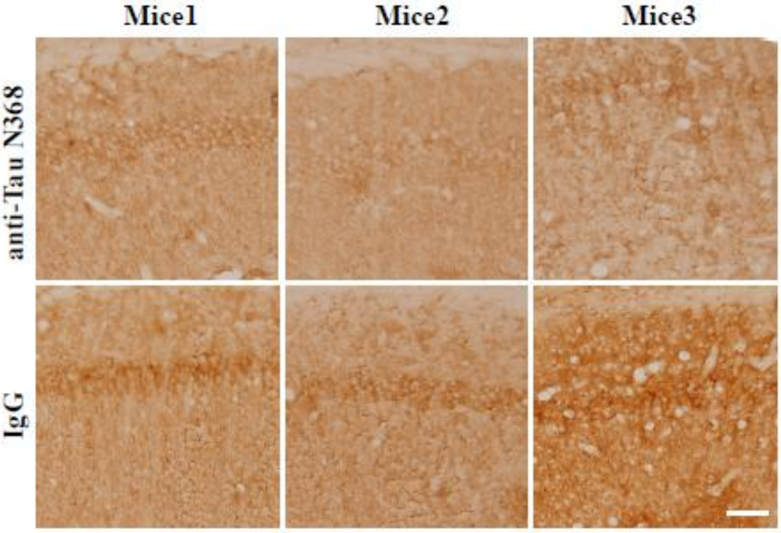

Cortex region Tau N368

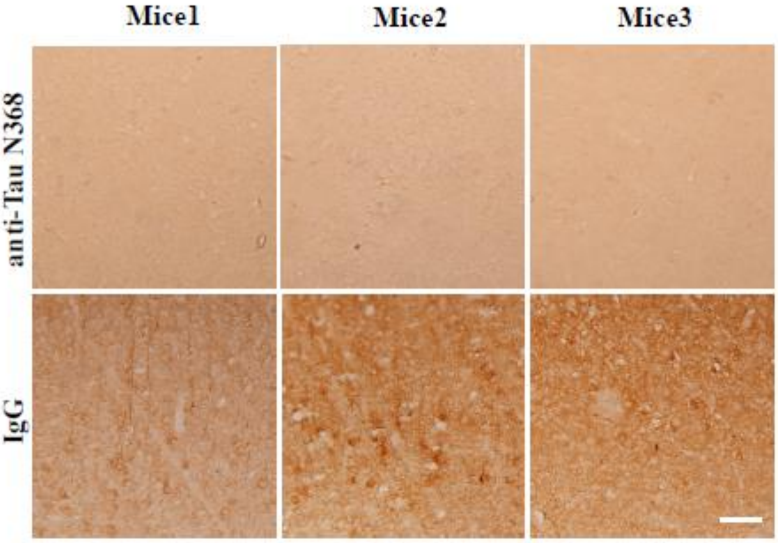

Extra Added P301S mice brain samples for Tau immunostaining

CA1 region AT8

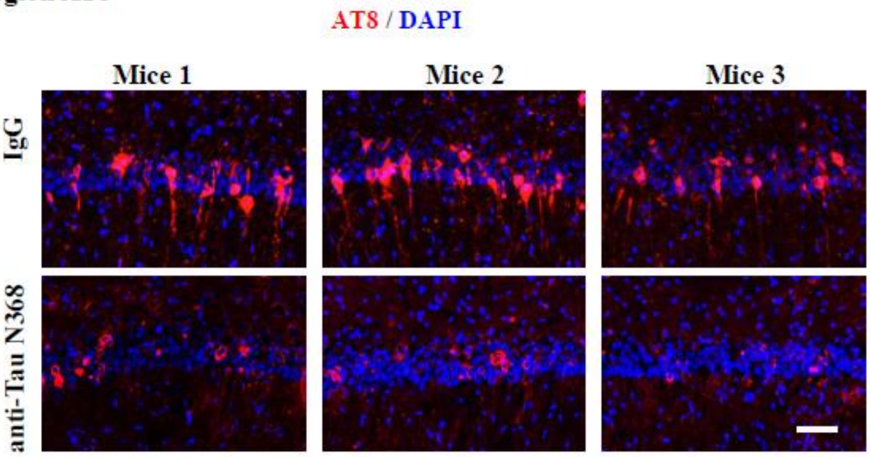

Cortex region AT8

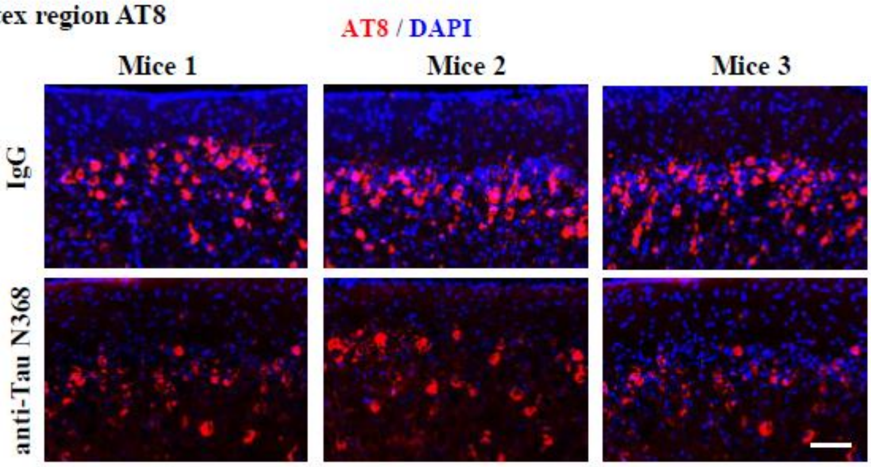

Extra Added P301S mice brain samples for Tau immunostaining

CA1 region Tau N368

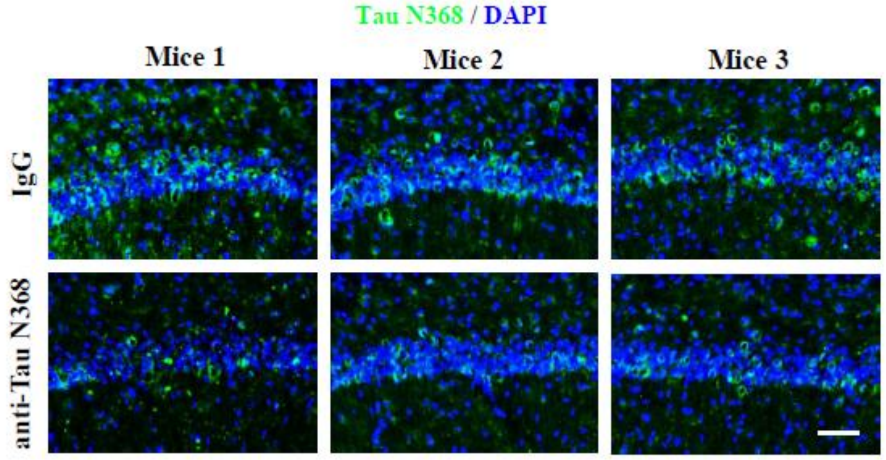

Cortex region Tau N368

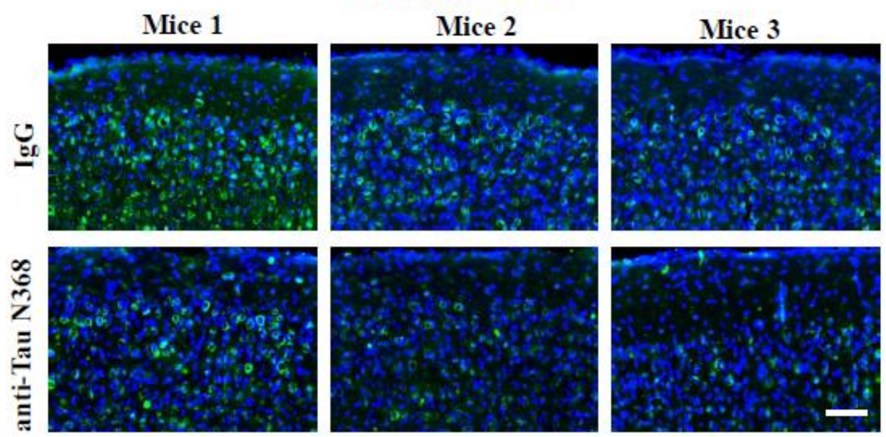

Extra Added P301S mice brain samples for Tau immunostaining

CA1 region AT100

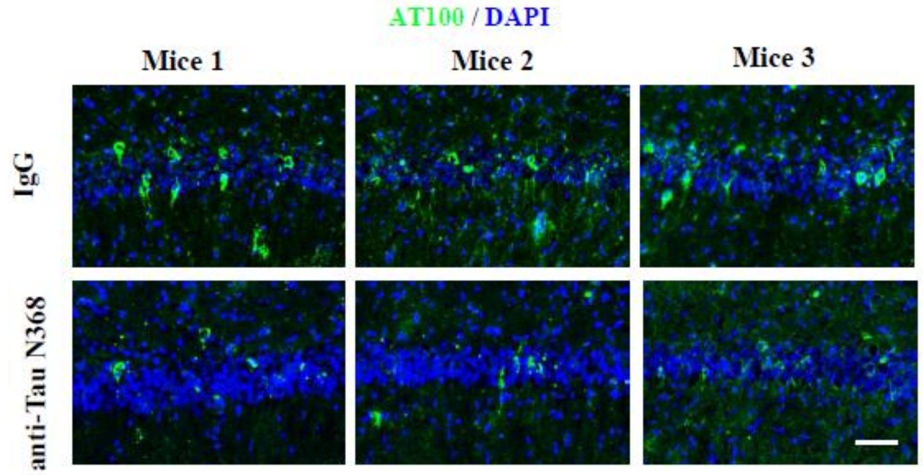

Cortex region AT100

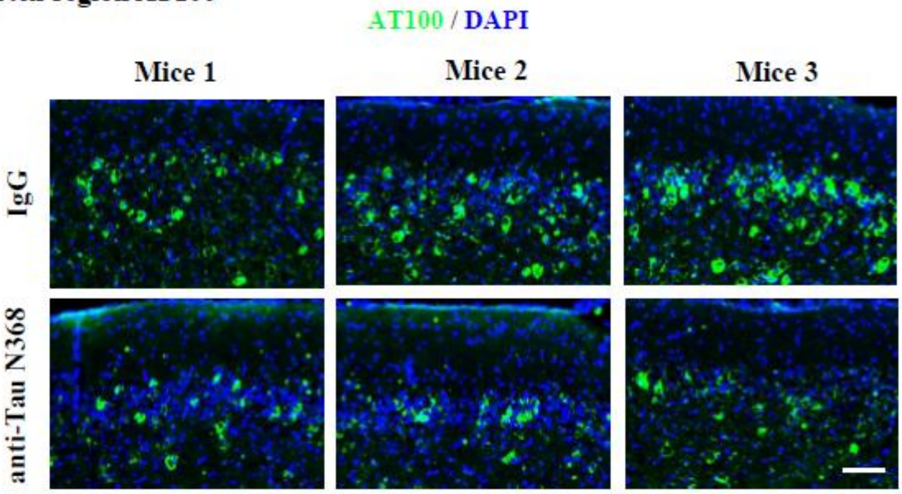

Supplement: Supplementary file 3 — Supplementary Material 3. [file 13024_2025_854_MOESM3_ESM.pdf]
