# Supplementary material for: Immunotherapy against tau fragment diminishes AD pathology, improving synaptic function and cognition
Source: Mol Neurodegener. 2025 May 27;20:60. doi: 10.1186/s13024-025-00854-9 (PMC12117789; doi:10.1186/s13024-025-00854-9)
Supplement: Supplementary file 4 — Supplementary Material 4. [file 13024_2025_854_MOESM4_ESM.pdf]

Fig. 1D-E

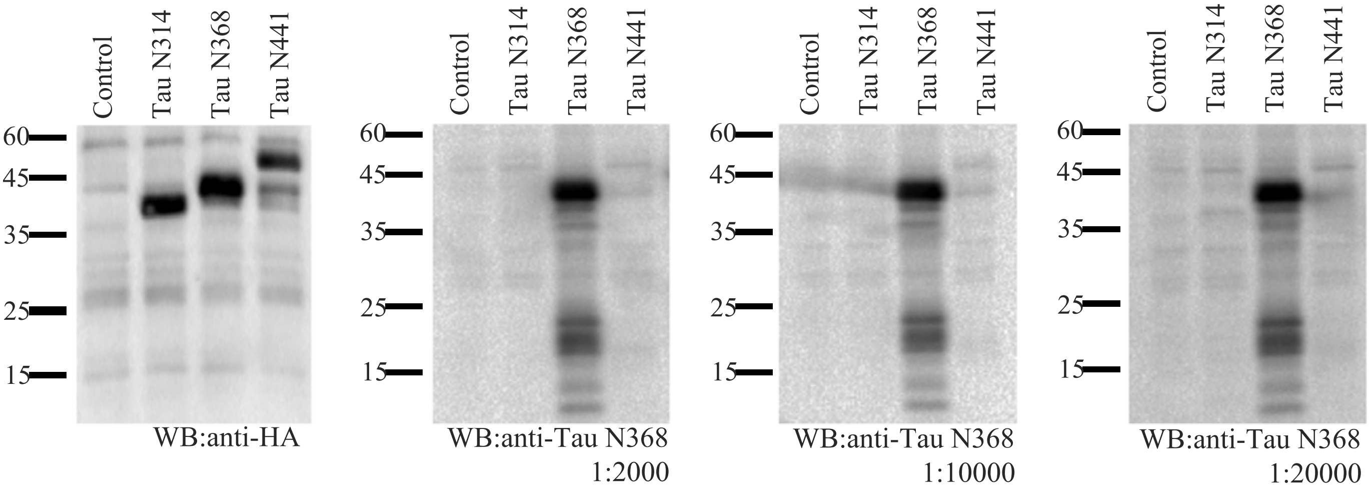

Control
Tau N314
Tau N368
Tau N441
60
45
35
25
15
WB:anti-HA
WB:anti-Tau N368
1:2000
WB:anti-Tau N368
1:10000
WB:anti-Tau N368
1:20000

Fig. 2B

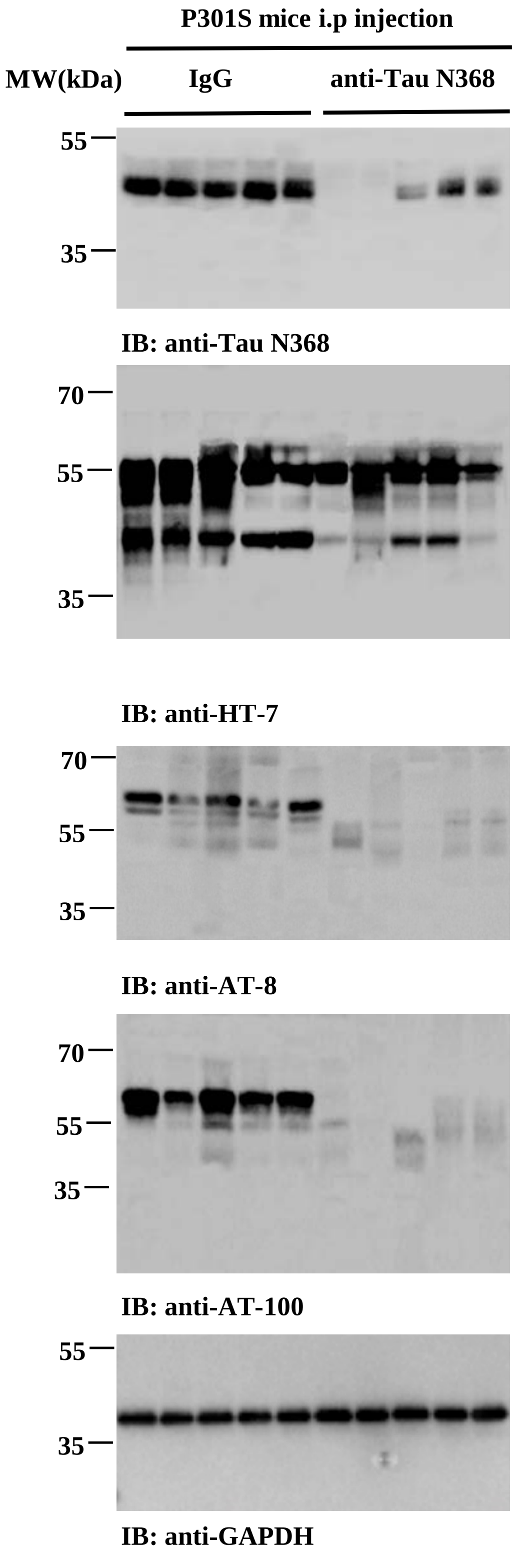

P301S mice i.p injection
MW(kDa)
IgG
anti-Tau N368
55
35
IB: anti-Tau N368
70
55
35
IB: anti-HT-7
70
55
35
IB: anti-AT-8
70
55
35
IB: anti-AT-100
55
35
IB: anti-GAPDH

Fig. 3F

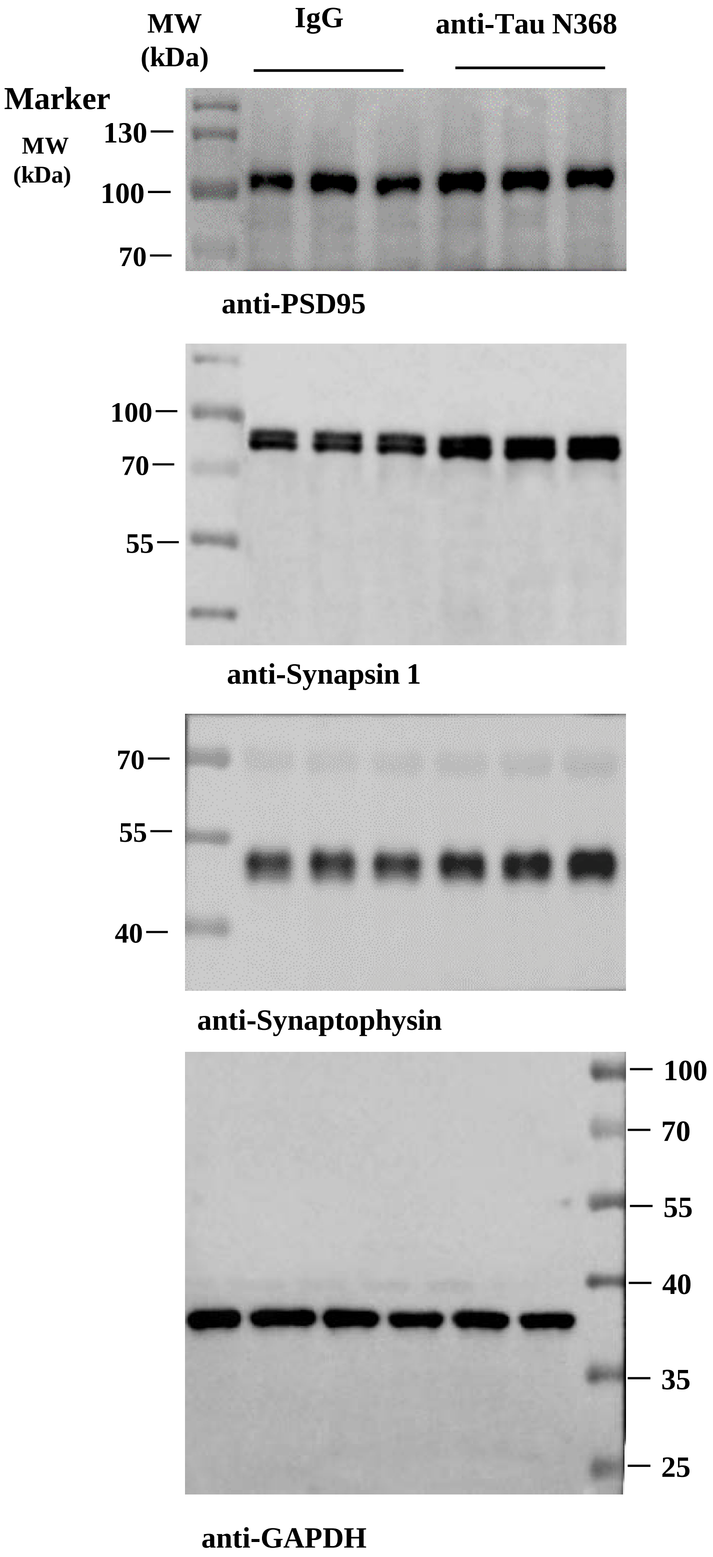
MW (kDa)
IgG
anti-Tau N368
Marker
MW (kDa)
130
100
70
anti-PSD95
100
70
55
anti-Synapsin 1
70
55
40
anti-Synaptophysin
100
70
55
40
35
25
anti-GAPDH

Fig. 4A

anti-TauN368

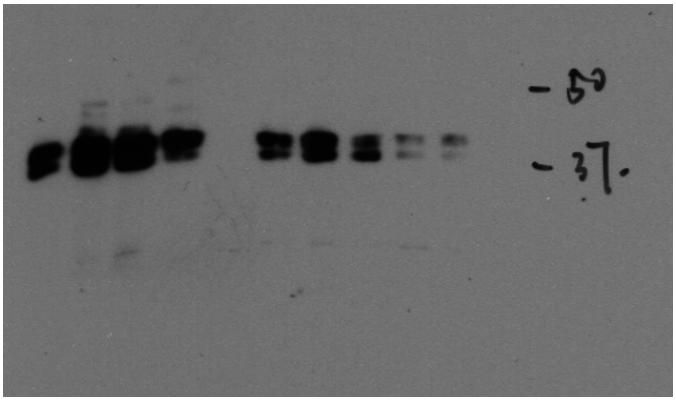

anti-AT8

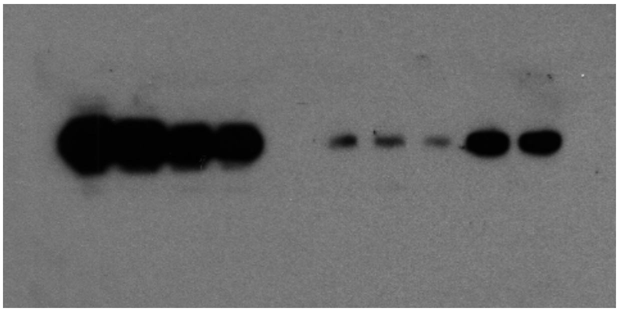

anti-HT7

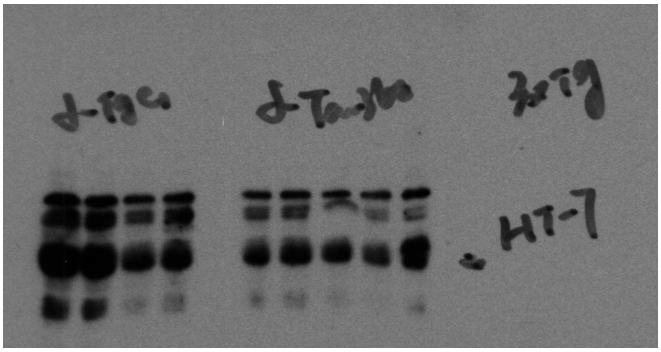

anti-beta-actin

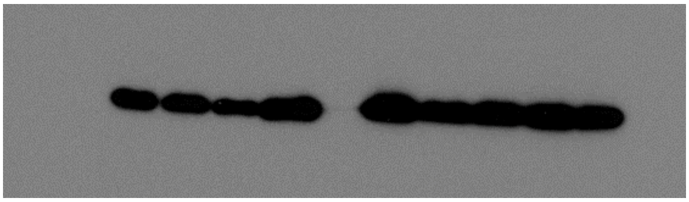

Fig. 6

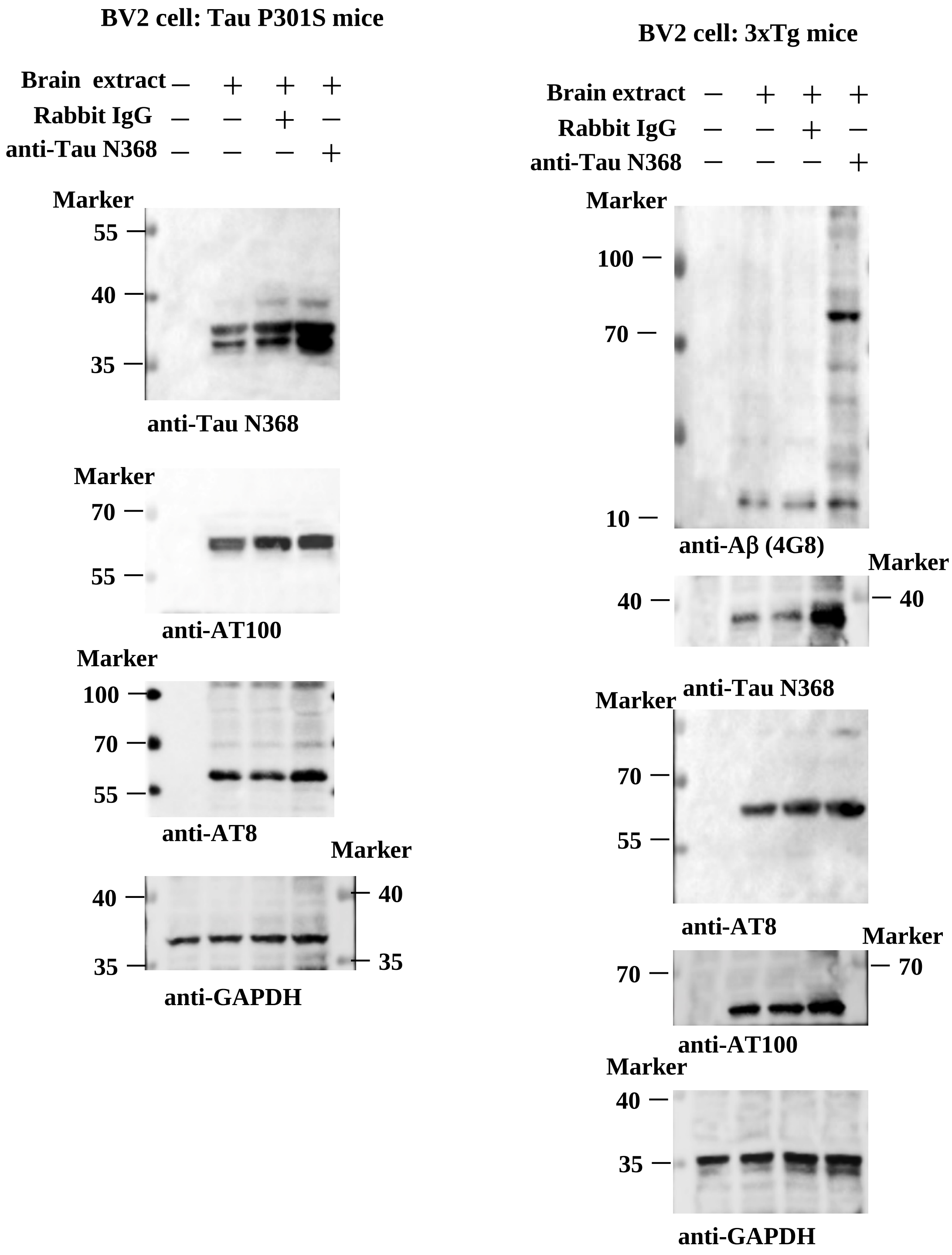
BV2 cell: Tau P301S mice
Brain extract – + + +
Rabbit IgG – – + –
anti-Tau N368 – – – +
Marker
55
40
35
anti-Tau N368
Marker
70
55
anti-AT100
Marker
100
70
55
anti-AT8
Marker
40
35
40
35
anti-GAPDH
BV2 cell: 3xTg mice
Brain extract – + + +
Rabbit IgG – – + –
anti-Tau N368 – – – +
Marker
100
70
10
anti-Aβ (4G8)
Marker
40
40
anti-Tau N368
Marker
70
55
anti-AT8
Marker
70
70
anti-AT100
Marker
40
35
anti-GAPDH

Fig. S1A

anti-Tau N368

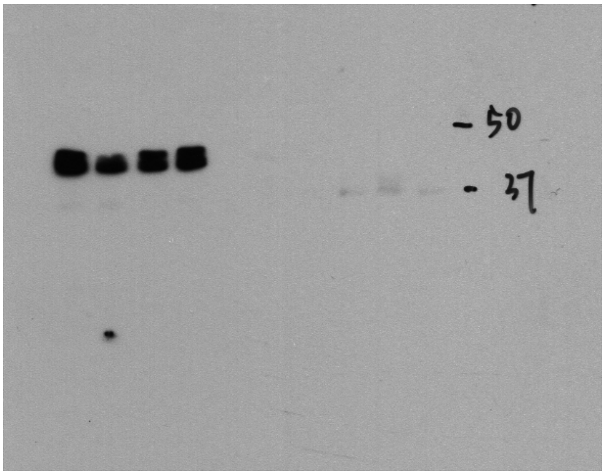

anti-HT7

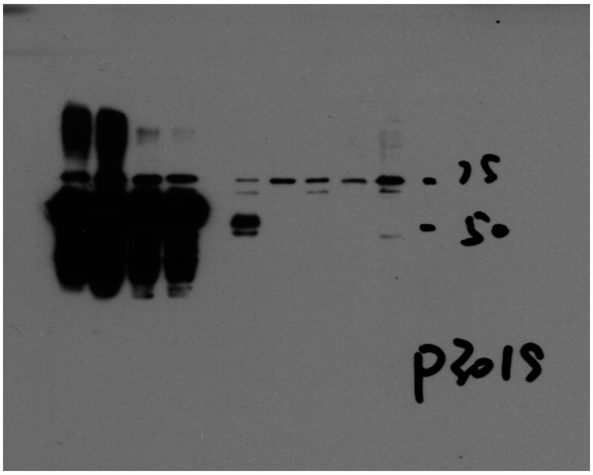

anti-AT-8

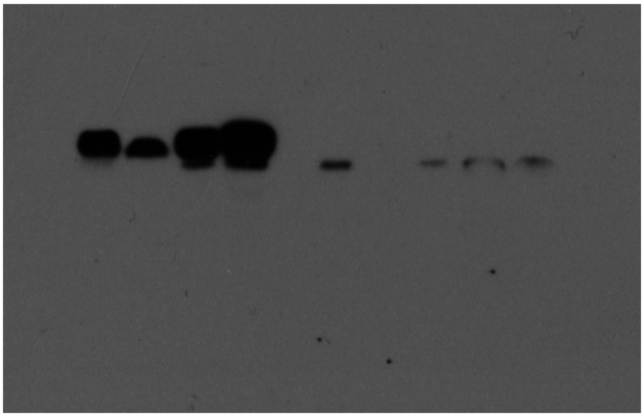

anti-AT-100

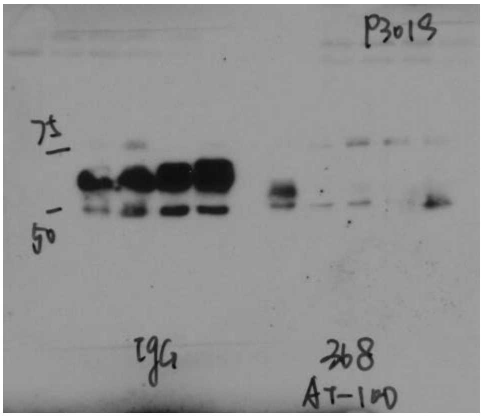

anti-GAPDH

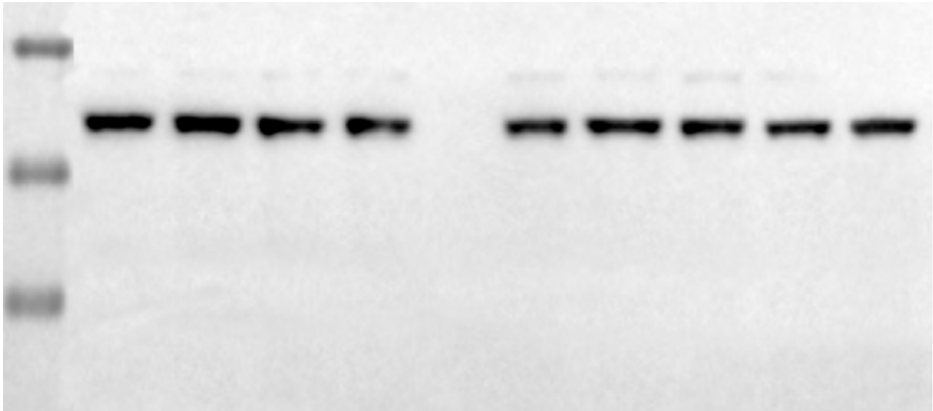

Fig. S3D

anti-pTrkB 816

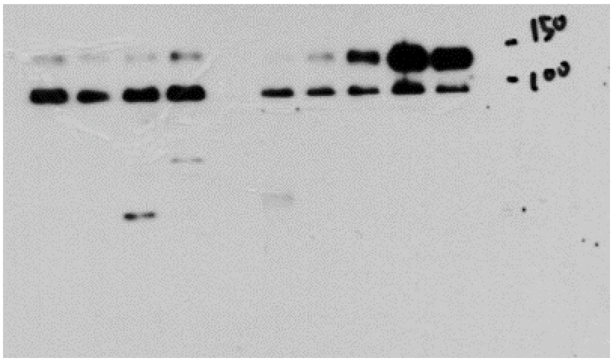

anti-TrkB

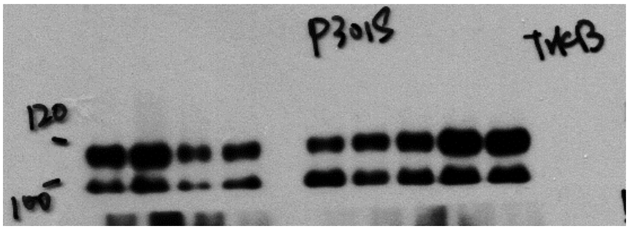

anti-pAKT

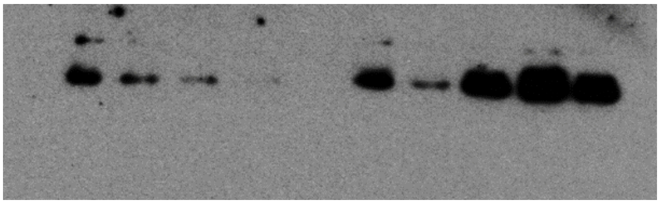

anti-AKT

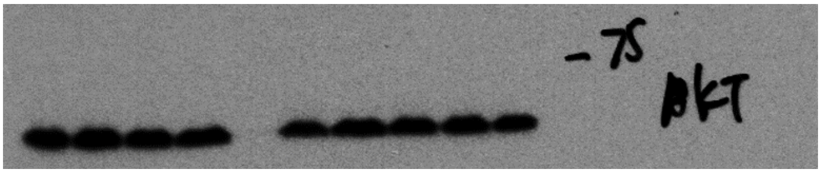

anti-pErk

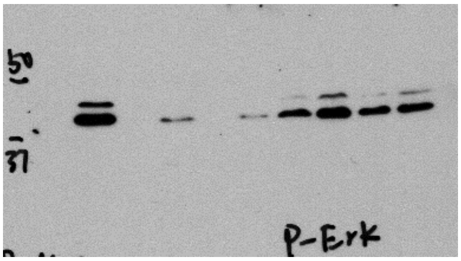

anti-Erk

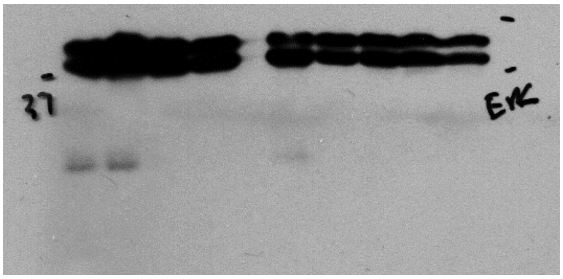

anti-beta-actin

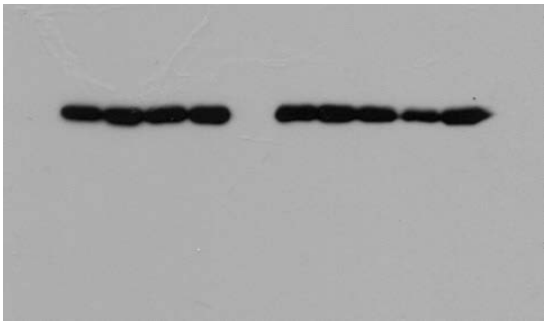

Original images of WB in Fig.S5

A **Tau P301S mice**

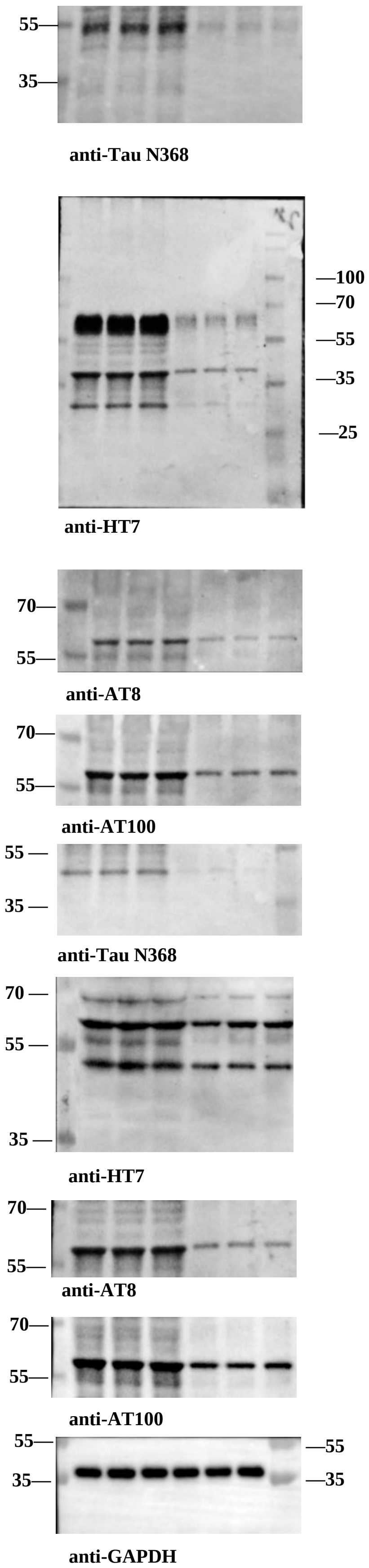

C **3xTg mice**

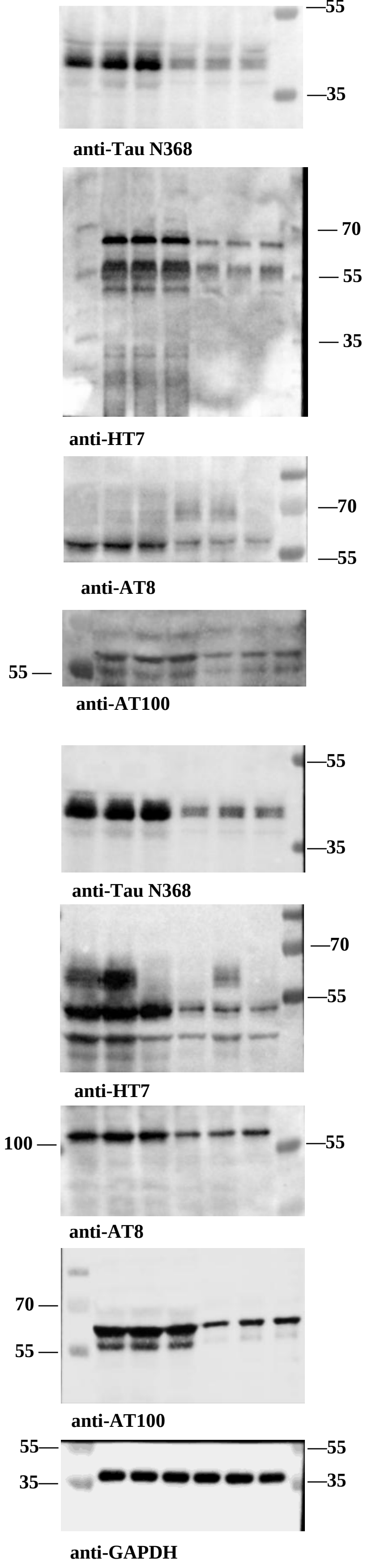

Fig. S6B

anti-pTrkB 816

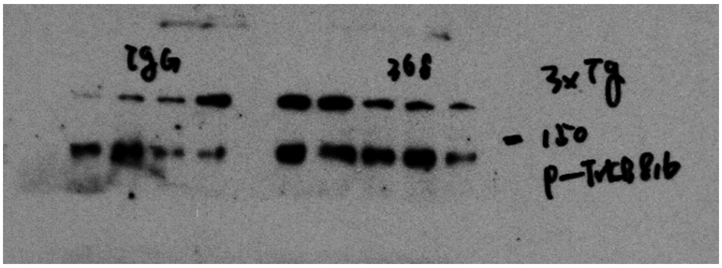

anti-TrkB

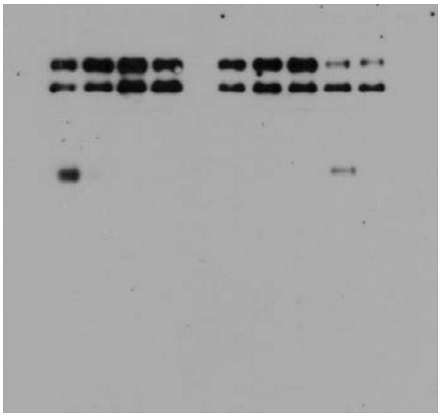

anti-pAKT

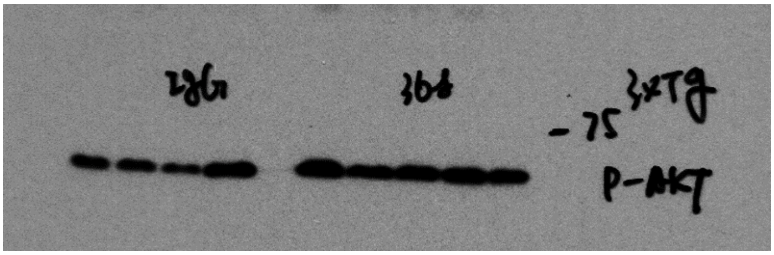

anti-AKT

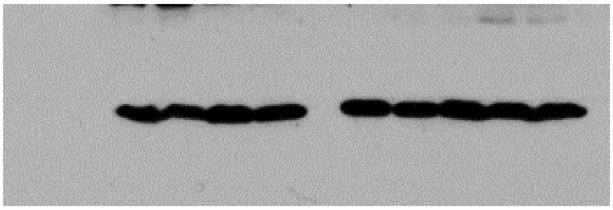

anti-pErk

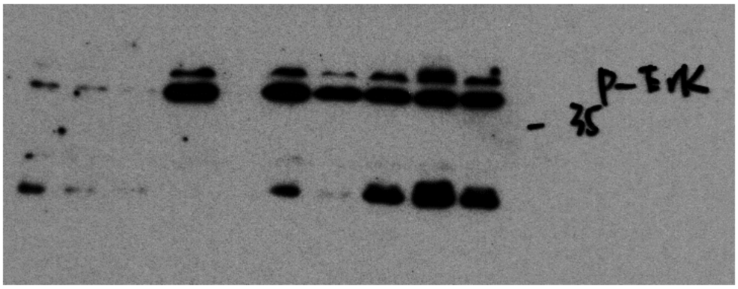

anti-Erk

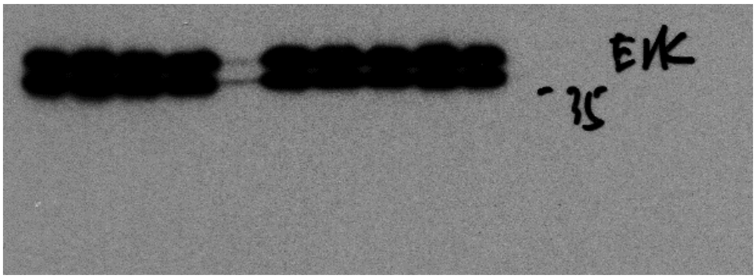

anti-beta-actin

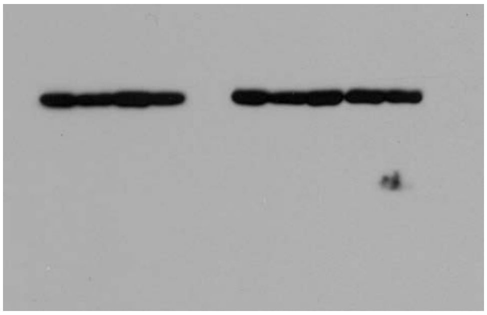

Fig. S9 A

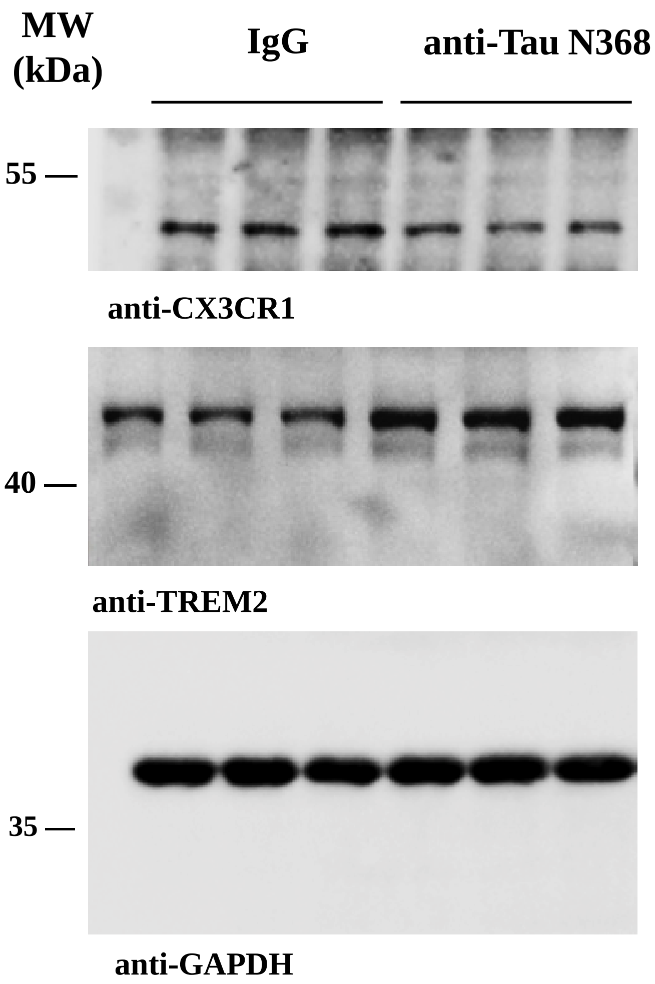

MW
(kDa)
IgG
anti-Tau N368
55 —
anti-CX3CR1
40 —
anti-TREM2
35 —
anti-GAPDH
