# Supplementary material for: Immunotherapy against tau fragment diminishes AD pathology, improving synaptic function and cognition
Source: Mol Neurodegener. 2025 May 27;20:60. doi: 10.1186/s13024-025-00854-9 (PMC12117789; doi:10.1186/s13024-025-00854-9)
Supplement: Supplementary file 5 — Supplementary Material 5. [file 13024_2025_854_MOESM5_ESM.pdf]

Supplementary materials

Fig. S1

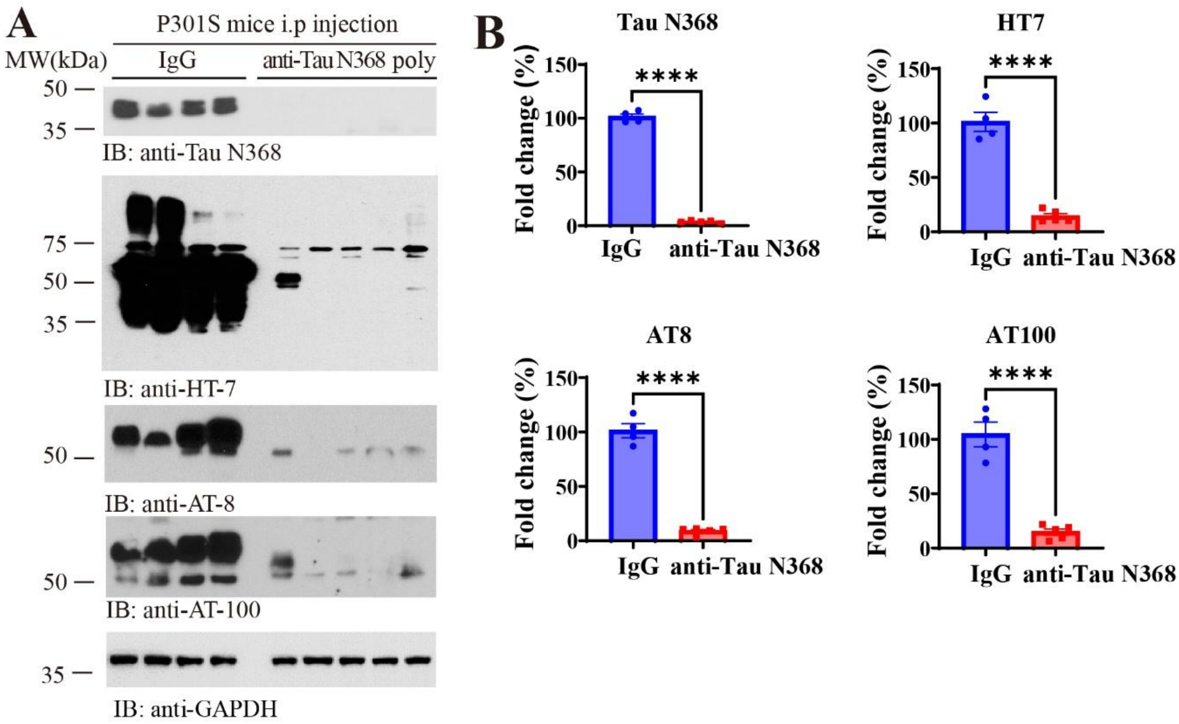

Fig S1 (former Fig 2 B-C) Anti-Tau N368 polyclonal antibody treatment inhibits Tau pathology in Tau P301S mice.

A. Representative immunoblot images showing the effects of anti-Tau N368 polyclonal antibody treatment on the expression of soluble Tau pathology-related proteins in the brains of Tau P301S mice with or without anti-Tau N368 treatment.

B. Relative quantification of the protein levels in (**A**). The data are presented as the means ± s.e.m.s; 4 or 5 mice in each group; *** $p < 0.001$, two-tailed Student's t test compared with IgG.

Fig. S2

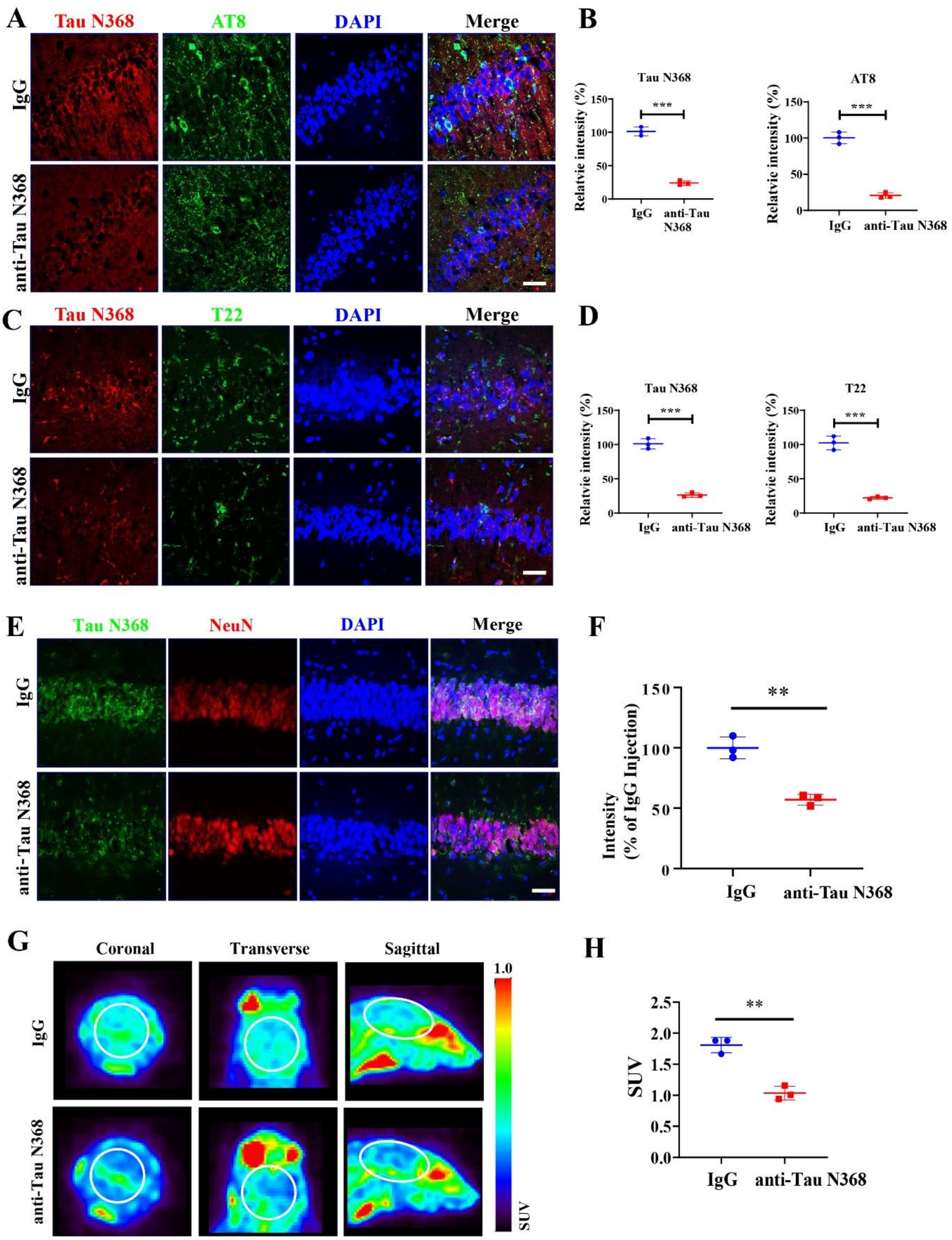
A
Tau N368
AT8
DAPI
Merge
IgG
anti-Tau N368
B
Tau N368
AT8
Relatvie intensity (%)
150
100
50
0
***
IgG
anti-Tau N368
C
Tau N368
T22
DAPI
Merge
IgG
anti-Tau N368
D
Tau N368
T22
Relatvie intensity (%)
***
IgG
anti-Tau N368
E
Tau N368
NeuN
DAPI
Merge
IgG
anti-Tau N368
F
Intensity (% of IgG Injection)
150
100
50
0
**
IgG
anti-Tau N368
G
Coronal
Transverse
Sagittal
IgG
anti-Tau N368
1.0
SUV
H
SUV
2.5
2.0
1.5
1.0
0.5
0.0
**
IgG
anti-Tau N368

Fig. S2 (former Fig S1) anti-Tau N368 treatment inhibits Tau phosphorylation and aggregation in Tau P301S mouse.

A. Representative immunofluorescence co-staining of Tau N368 and AT8 in the hippocampus of Tau P301S mice with or without anti-Tau N368 treatment. Scale bar, 20 μm.

B. Quantification of AT8 and Tau N368 fluorescent signals in (**A**). *** $p < 0.001$, compared with IgG, two-tailed Student's t-test. The data are presented as the means ± s.e.m.; n=3 mice in each group.

C. Representative immunofluorescence co-staining of Tau N368 and T22 in the hippocampus of Tau P301S mice with or without anti-Tau N368 treatment. Scale bar, 20 μm.

D. Quantification of T22 and Tau N368 fluorescent signals in (**C**). *** $p < 0.001$, compared with IgG, two-tailed Student's t-test. The data are presented as the means ± s.e.m.; n=3 mice in each group.

D. Representative immunofluorescent images of Tau N368 in the hippocampus region by anti-rabbit polyclonal Tau N368 antibody. Scale bar, 20 μm.

E. Quantification of fluorescent signal intensity in A. (n = 3 mice, **$p < 0.01$, compared with IgG treatment). The data are presented as the means ± s.e.m.; n=3 mice in each group.

F. Tau PET images showing the tau deposition in the brains of Tau P301S mouse. The brain region was labeled with the white circle. Tau PET images showing the Tau deposition in the brains of P301S mice.

G. Relative quantification of Tau PET standard uptake (SUV) in A (n = 3 mice, **$p < 0.01$, compared with IgG treatment). The data are presented as the means ± s.e.m.; n=3 mice in each group.

Fig. S3

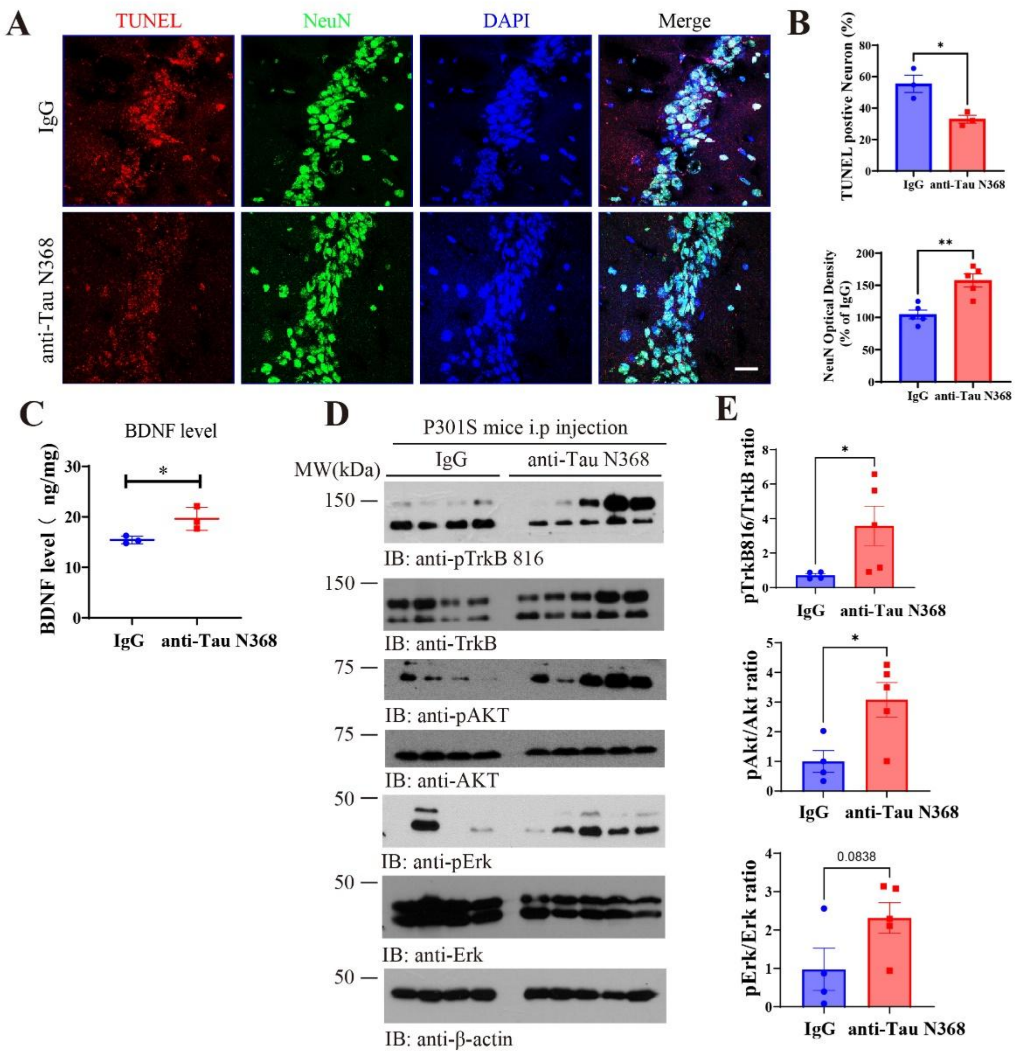

Fig. S3 (former Fig S2) Anti-Tau N368 treatment ameliorating neuronal loss in the hippocampus by rescuing TrkB signaling.

A. Immunofluorescence staining of NeuN and TUNEL in the hippocampus of Tau P301S mice with or without anti-Tau N368 treatment. Scale bar, 20 μm.

B. Relative quantification of NeuN and TUNEL fluorescence intensity in (**A**). n = 3, * $p < 0.05$, compared with IgG. two-tailed Student's t-test. The data are presented as the means ± s.e.m.; n=3 mice in each group.

C. ELISA analysis of BDNF in brain tissue of Tau P301S mice with or without anti-Tau N368 treatment. * $p < 0.05$, compared with IgG. Two-tailed Student's t-test. The data are presented as the means ± s.e.m.; n=3 mice in each group.

D. Representative immunoblot images showing the effects of anti-Tau N368 treatment on TrkB signaling pathway in the brains of Tau P301S mice with or without anti-Tau N368 treatment.

E. Relative quantification of protein levels in (**D**). * $p < 0.05$, *** $p < 0.001$ compared with IgG. two-tailed Student's t-test. The data are presented as the means ± s.e.m.; n=4 (IgG) or n=5 (anti-Tau N368) mice in each group.

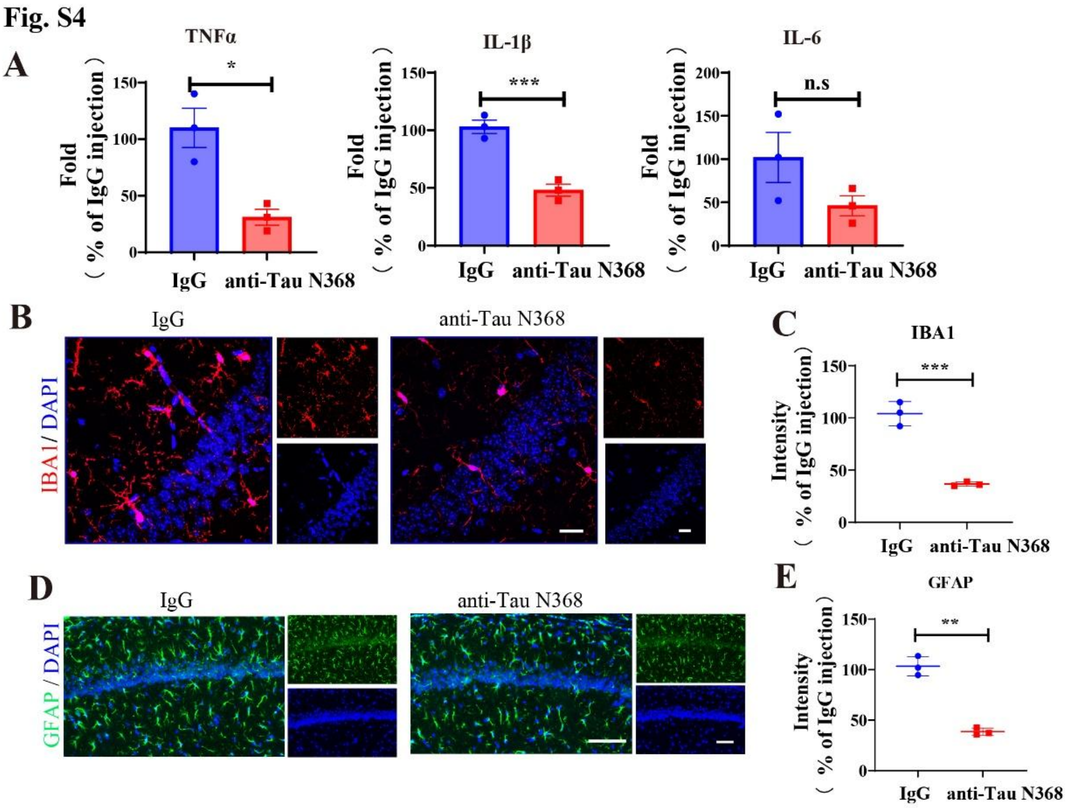

Fig. S4 Anti-Tau N368 treatment inhibits neuro-inflammation in Tau P301S mouse.

A. ELISA analysis of inflammatory cytokines in brain tissue of Tau P301S mice with or without anti-Tau N368 treatment. * $p < 0.05$, *** $p < 0.001$ compared with IgG. two-tailed Student's t-test. n.s., no significant. The data are presented as the means ± s.e.m.; n=3 mice in each group.

B. Representative immunofluorescence staining of IBA1 in the hippocampus of Tau P301S mice with or without anti-Tau N368 treatment. Scale bar, 20 μm. The data are presented as the means ± s.e.m.; n=3 mice in each group.

C. Relative quantification of IBA1 fluorescence intensity in (**B**). *** $p < 0.001$ compared with IgG, two-tailed Student's t-test. The data are presented as the means ± s.e.m.; n=3 mice in each group.

D. Immunofluorescence staining of GFAP in the hippocampus of Tau P301S mice with or without anti-Tau N368 treatment. Scale bar, 100 μm.

E. Relative quantification of GFAP fluorescence intensities in (**D**). ** $p < 0.01$, compared with IgG, two-tailed Student's t-test. The data are presented as the means ± s.e.m.; n=3 mice in each group.

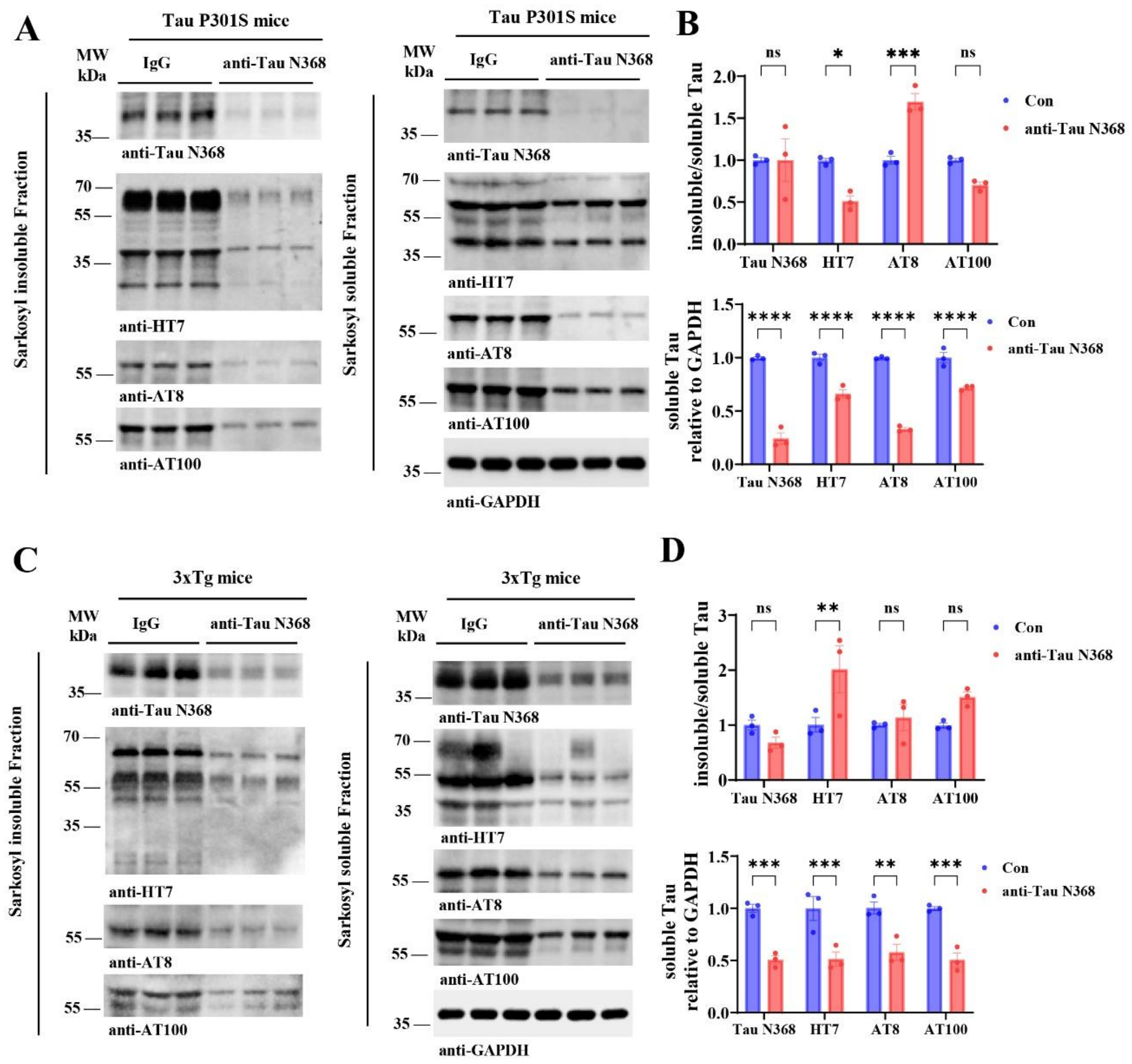

Fig. S5 (newly added) Anti-Tau N368 treatment reduced both sarkosyl soluble and insoluble tau in P301S and 3xTg brain.

A-B. Immunoblot images and quantifications of Tau N368, AT8, AT100 and total Tau in sarkosyl soluble and insoluble fractions from P301S brain of treated anti-Tau N368. n=3 Mice per group. The data are presented as the means ± s.e.m. Statistical significance were determined by two-way ANOVA and with posthoc Sidak's test. * $p < 0.05$, ** $p < 0.01$, *** $p < 0.001$, ns=no significance.

C-D. Immunoblot images and quantifications of Tau N368, AT8, AT100 and total Tau in sarkosyl soluble and insoluble fractions from 3xTg brain of treated anti-Tau N368. n=3 Mice

per group. The data are presented as the means ± s.e.m. Statistical significance were determined by two-way ANOVA and with posthoc Sidak's test. * $p < 0.05$, ** $p < 0.01$, *** $p < 0.001$, ns=no significance.

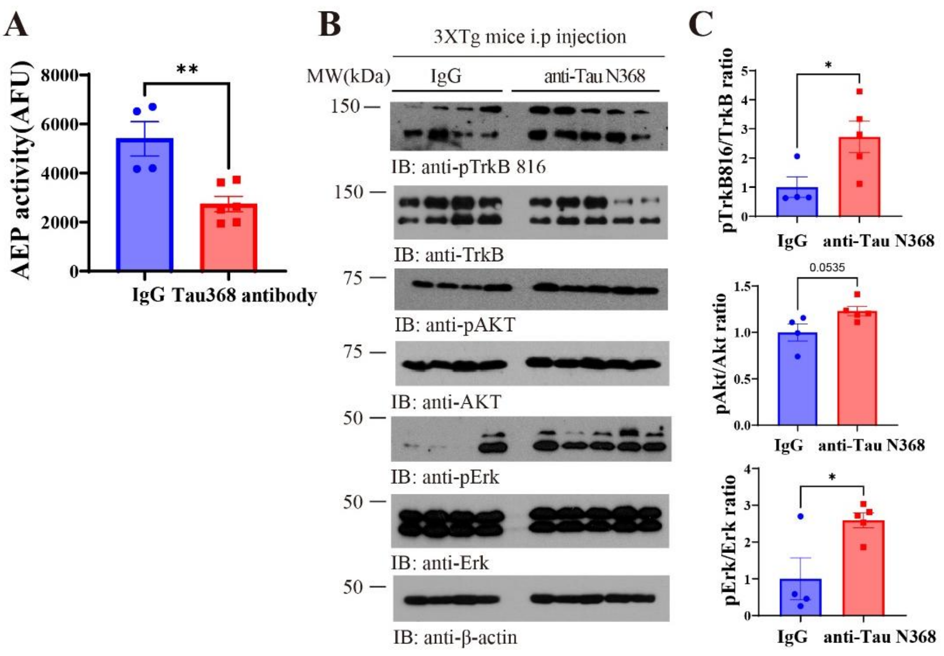

Fig. S6 (former Fig S5) Anti-Tau N368 treatment rescued TrkB signaling and inhibited AEP activity.

A. AEP enzymatic activity in brain tissues of 3xTg mice with or without anti-Tau N368 treatment. ** $p < 0.01$, compared with IgG.The data are presented as the means ± s.e.m.; n=4 (IgG) or n=6 (anti-Tau N368) mice in each group.

B. Representative immunoblot images showing the effects of anti-Tau N368 treatment on TrkB signaling pathway in the brains of 3xTg mice with or without anti-Tau N368 treatment.

C. Relative quantification of protein levels in (**B**). * $p < 0.05$, ** $p < 0.01$, *** $p < 0.001$ compared with IgG. The data are presented as the means ± s.e.m.; n=4 (IgG) or n=5 (anti-Tau N368) mice in each group.

Fig. S7

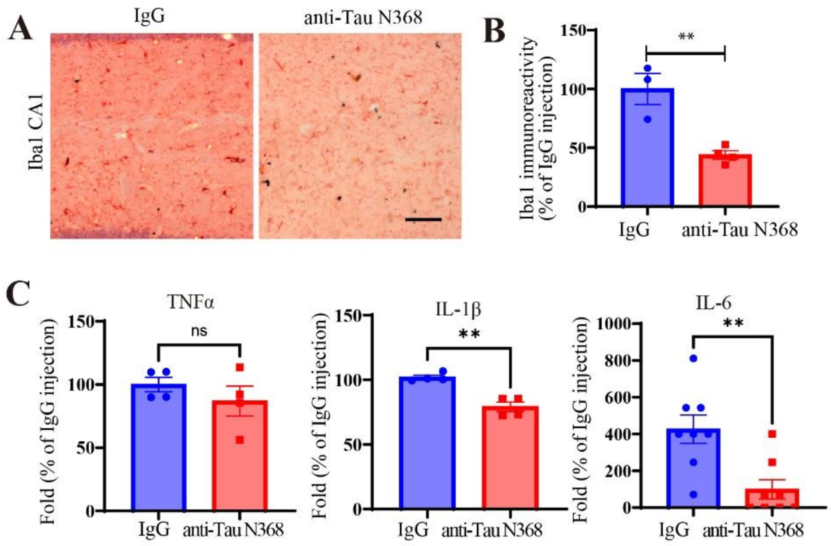

Fig. S7 (former Fig S6) Anti-Tau N368 treatment inhibits neuroinflammation in 3xTg mouse.

A. Representative immunohistochemistry (IHC) staining image of IBA1 in hippocampus CA1 region of 3xTg mice with or without anti-Tau N368 treatment. Scale bar, 20 μm.

B. Relative quantification of IBA1 signal intensity in (**A**). n = 3 (IgG) and n=4 (anti-Tau N368), ** $p < 0.01$, compared with IgG.

C. ELISA analysis of inflammatory cytokines in the brain tissues of 3xTg mice with or without anti-Tau N368 treatment. ** $p < 0.01$, compared with IgG. n.s., no significant. The data are presented as the means ± s.e.m.; n=4 mice in TNFα and IL-1β of group. n=8 mice in IL-6 group.

Fig. S8

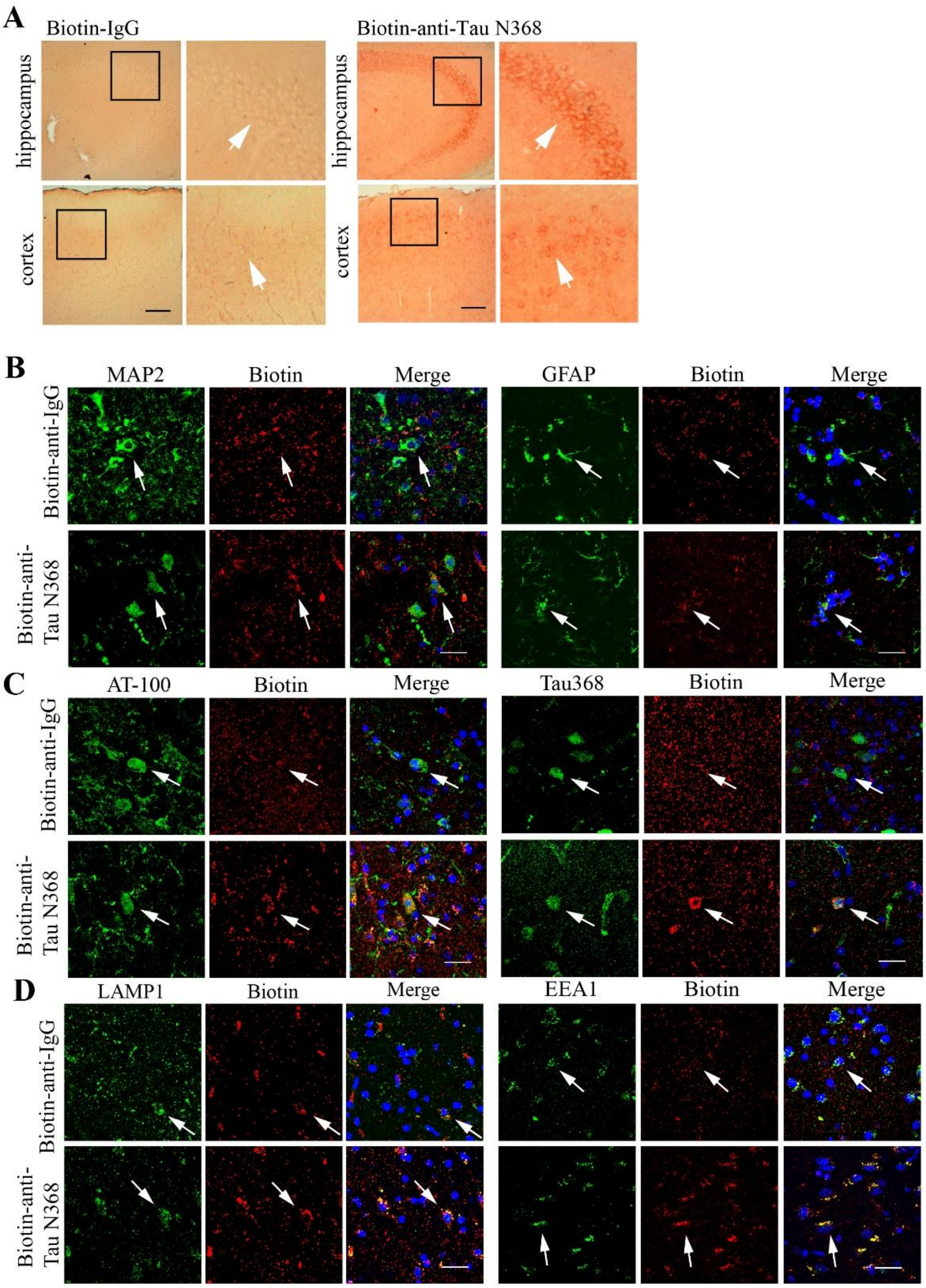

A
Biotin-IgG
Biotin-anti-Tau N368
hippocampus
cortex
hippocampus
cortex
B
MAP2
Biotin
Merge
GFAP
Biotin
Merge
Biotin-anti-IgG
Biotin-anti-Tau N368
C
AT-100
Biotin
Merge
Tau368
Biotin
Merge
Biotin-anti-IgG
Biotin-anti-Tau N368
D
LAMP1
Biotin
Merge
EEA1
Biotin
Merge
Biotin-anti-IgG
Biotin-anti-Tau N368

Fig. S8（former Fig S7） Tau N368 antibody penetrated the brain and eliminated the intracellular pathologic Tau N368.

A. Tau N368 antibody transported into the brain region after i.p. administration. After i.p. injection of biotinylated Tau N368 antibody for 24 hours, IHC staining was conducted on the brain sections of P301S mice or WT mice. CA1 IHC staining was shown with streptavidin-HRP antibody (arrows). The right panels show higher magnification of the left boxed region: scale bar, 50 μm. n=4 mice per group.

B-D. Tau N368 antibody penetrated the neurons in cultured organotypic hippocampal slices. Immunofluorescent co-staining with biotinylated-Tau N368 antibody (Red) and other antibodies (Green) were conducted (arrows). n=3 mice per group.

B. Immunofluorescent co-staining with biotinylated-Tau N368 antibody (Red) and neuron (MAP2) or astrocytes (GFAP) markers (Green). (Scale bar, 10 μm).

C. Immunofluorescent co-staining with biotinylated-Tau N368 antibody (Red) and phosphorylated Tau (AT-100) or TauN368. (Scale bar, 10 μm).

D. Immunofluorescent co-staining with biotinylated-Tau N368 antibody (Red) and lysosome (LAMP1) or early endosome (EEA1) markers. (Scale bar, 10 μm).

Fig. S9

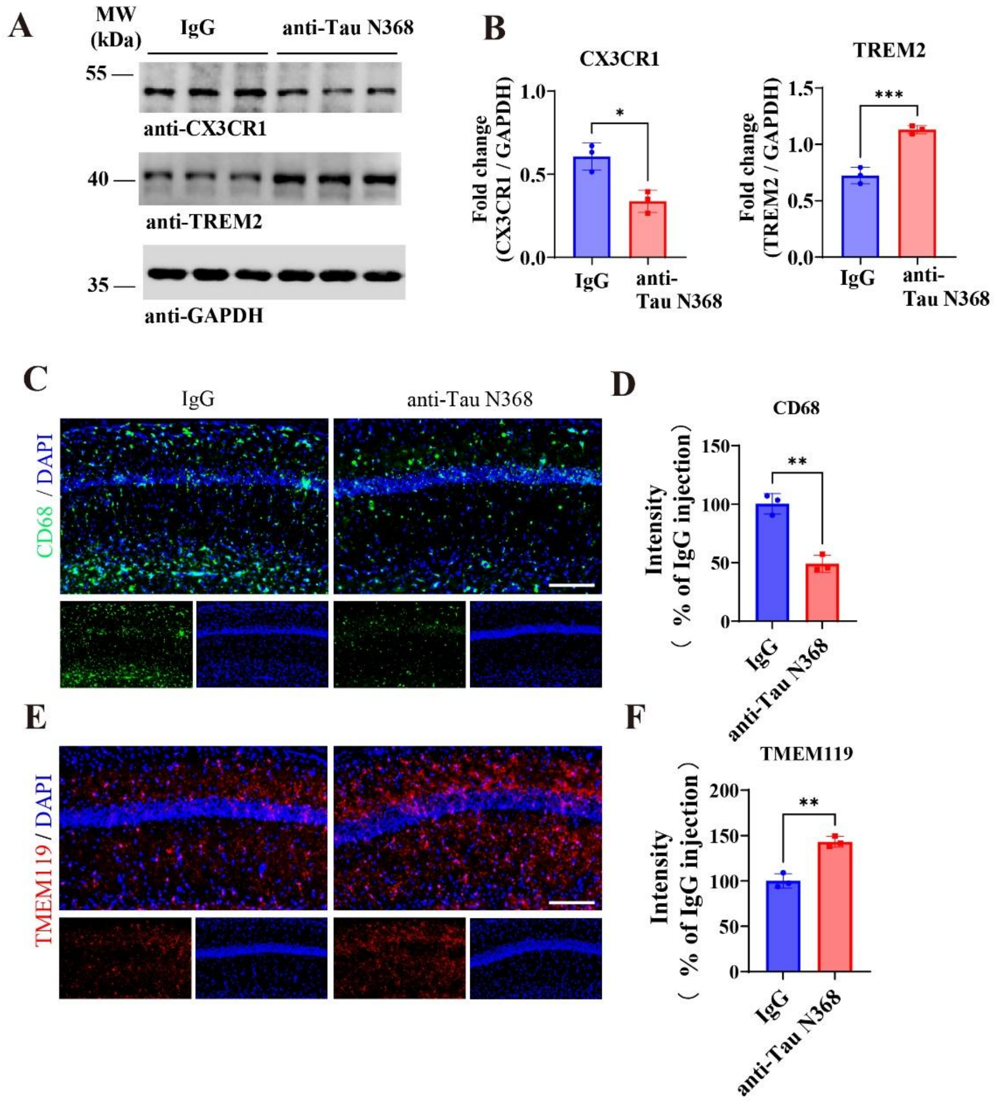

Fig S9(Former S8). Anti-Tau N368 treatment activates microglia phagocytic functions in the brain of Tau P301S mice.

A. Representative western blot images of the microglial marker in response to anti-Tau N368.

B. Relative quantification of A. n=3, ***p <0.001, *p < 0.05, Student's t-test. The data are presented as the means ± s.e.m.; n=3 mice in each group.

C-F， Representative images of CD68 (**C**) and TMEM119 (**E**) immunofluorescent staining in hippocampus and their quantification (**D & F**). Scale bar, 100 μm. ** p < 0.01, compared with

IgG, two-tailed Student's t-test. The data are presented as the means ± s.e.m.; n=3 mice in each group.
